# MYB regulates the SUMO protease SENP1 and its novel interaction partner UXT, modulating MYB target genes and the SUMO landscape

**DOI:** 10.1016/j.jbc.2023.105062

**Published:** 2023-07-17

**Authors:** Roza Berhanu Lemma, Marit Ledsaak, Bettina Maria Fuglerud, Fernando Rodríguez-Castañeda, Ragnhild Eskeland, Odd Stokke Gabrielsen

**Affiliations:** 1 Department of Biosciences, University of Oslo, Oslo, Norway; 2 Centre for Molecular Medicine Norway (NCMM), Nordic EMBL Partnership, University of Oslo, Oslo, Norway; 3 Faculty of Medicine, Department of Molecular Medicine, Institute of Basic Medical Sciences, University of Oslo, Oslo, Norway; 4 Faculty of Medicine, Centre for Cancer Cell Reprogramming, Institute of Clinical Medicine, University of Oslo, Oslo, Norway

**Keywords:** MYB, pioneer transcription factor, SENP1, SUMO, UXT, auto-activation, gene regulation

## Abstract

SUMOylation is a post-translational modification frequently found on nuclear proteins, including transcription factors (TFs) and coactivators. By controlling the activity of several TFs, SUMOylation may have far-reaching effects. MYB is an example of a developmental TF subjected to SUMO-mediated regulation, through both SUMO conjugation and SUMO binding. How SUMO affects MYB target genes is unknown. Here, we explored the global effect of reduced SUMOylation of MYB on its downstream gene programs. RNA-Seq in K562 cells after MYB knockdown and rescue with mutants having an altered SUMO status revealed a number of differentially regulated genes and distinct gene ontology term enrichments. Clearly, the SUMO status of MYB both quantitatively and qualitatively affects its regulome. The transcriptome data further revealed that MYB upregulates the SUMO protease SENP1, a key enzyme that removes SUMO conjugation from SUMOylated proteins. Given this role of SENP1 in the MYB regulome, we expanded the analysis, mapped interaction partners of SENP1, and identified UXT as a novel player affecting the SUMO system by acting as a repressor of SENP1. MYB inhibits the expression of UXT suggesting that MYB is able not only to control a specific gene program directly but also indirectly by affecting the SUMO landscape through SENP1 and UXT. These findings suggest an autoactivation loop whereby MYB, through enhancing SENP1 and reducing UXT, is itself being activated by a reduced level of repressive SUMOylation. We propose that overexpressed MYB, seen in multiple cancers, may drive this autoactivation loop and contribute to oncogenic activation of MYB.

Small ubiquitin-like modifier proteins (SUMOs) are posttranslational modifiers that become covalently conjugated to lysine residues of their target proteins (1, 2, 3, 4, 5). In addition to covalent conjugations, SUMOs may also become noncovalently bound to SUMO interaction Motif (SIM)-containing proteins. SUMOylation is particularly prevalent in the nucleus where about 80% of the nuclear proteins are modified under standard conditions (6, 7, 8). Consequently, a wide range of nuclear processes are affected by SUMOylation, such as transcription, chromatin remodeling, pre-mRNA splicing, ribosome assembly, DNA repair, maintenance of genome integrity, nuclear transport, and signal transduction (reviewed in (7, 9, 10, 11, 12). Typically, 60 to 80% of all proteins involved in these processes are being modified by SUMOylation at some point (6). The potential for SUMO-mediated global regulation of cellular processes is therefore significant.

In the SUMO system, there is a balance between SUMOylation and deSUMOylation (3, 5). This balance is achieved with the help of SUMO-specific proteases that deconjugate SUMO from the target protein. These proteins in humans are referred to as sentrin-specific proteases (SENPs). There are six members of the SENP family in humans (SENP1-3 and SENP5-7) (5, 13). They are characterized by a conserved catalytic C-terminal domain and a variable N-terminal domain, which governs subcellular localization and substrate specificity (13). The multiplicity of SENPs adds to the regulatory landscape of nuclear processes.

The regulatory role of SUMOylation in transcription has traditionally been regarded as a negative control. When studied individually, the majority of transcription factors (TFs) were found to be impaired in their activation of target genes upon SUMOylation (14). This implies that significant gene activation may be obtained upon the relief of SUMO-mediated repression. However the mechanism of this derepression, mediated by SENPs, is poorly understood. We have previously suggested that an interplay between SENPs and chromatin remodeling may be one element in this mechanism (15). Globally, SUMOylation may also change the interaction repertoire of TFs and affect their ability to synergize with other factors and in some cases, even change their subcellular localization (16). A striking example is the glucocorticoid receptor, which, when bound to chromatin, will recruit a different set of chromatin modifiers dependent on its SUMOylation status (17). SUMO may be conjugated to many players involved in the same process through “group SUMOylation” (1, 18, 19). In addition, SUMO may also modulate the partitioning of its targets in liquid-liquid phase separation processes and thereby affect gene expression (20). The overall outcome of SUMOylation in transcription may therefore be complex.

MYB is a sequence-specific TF subjected to both SUMO conjugation and SUMO binding (21, 22, 23, 24, 25). It is a key regulator of stem and progenitor cells in the bone marrow, colonic crypts, and a neurogenic region of the adult brain (26, 27). In the hematopoietic system, MYB is essential for proper development (27, 28, 29), where *c-myb*^−/−^ mice are embryonic lethal due to a failure in fetal liver hematopoiesis (30). MYB is required for the development of both myeloid and lymphoid progenitor cells (28, 31) and is considered a developmental regulator because of its role in lineage determination and its control of the activity of other transcriptional regulators (32, 33, 34). Recently, specific novel functions of MYB in the T-cell compartment have been defined. MYB was reported to be essential for generating and maintaining stem cells in the CD8+ T-cell memory compartment (35). Furthermore, MYB was found to be essential for the development and function of a specific subpopulation of T cells, namely CD62 L+ stem-like T cells, that is central to the maintenance of long-term antiviral immunity and responsiveness to immunotherapy (36). Mechanistically, MYB activates its target genes through DNA binding (37) and transactivation (38). In addition, MYB is involved in recruiting chromatin remodelers (39) and shows histone-binding properties (40, 41). We have recently shown through studies of a mutation with impaired histone binding that MYB does operate as a pioneer factor (42) in line with its role as a developmental regulator. MYB may also have a general role in super-enhancer initiation (33). How the SUMO status of MYB affect all these properties is poorly understood. One effect may be to restrict synergy between TFs and limiting their cooperation on complex promoters. We have previously reported that there is a strong synergy control linked to the SUMO conjugation status of MYB (24). Although a wide range of MYB target genes have been identified (42, 43, 44), little is known about how these are affected by alterations in SUMO conjugation or SUMO binding.

In this study, we investigated the interplay between MYB-dependent gene activation and SUMO status by two main lines of research. First, we asked how an altered SUMO status of MYB affected its target genes globally. In this first line of research, we performed RNA-Seq analysis after endogenous MYB knockdown and rescue using single cell clones stably expressing either WT MYB or one of two MYB SUMO mutants, a SUMO conjugation negative mutant and a SUMO-binding mutant. For reasons of simplicity, the transfected cell clones are subsequently referred to as rescue clones. Then, we observed that SENP1 is a direct target gene of MYB being upregulated by both WT- and SUMO-negative MYB rescues. In the second line of research, to better understand the role of SENP1 in the MYB regulome, we mapped interaction partners of SENP1 and identified ubiquitously expressed transcript (UXT) as a novel interaction partner of SENP1. We found that UXT functionally operates as an SENP1 inhibitor, both *in vitro* and *in vivo*. We also observed that UXT is a direct target gene of MYB but being downregulated by both the WT- and SUMO-negative MYB rescues. The opposite regulation of SENP1 and UXT by MYB suggests that MYB may modulate the SUMOylation landscape through activation of a SUMO protease and downregulation of its inhibitor. Since MYB itself is repressed by SUMOylation, this also suggests an autoactivation loop through its effect on SENP1 and UXT.

## Results

### How does the SUMO status of MYB affect its regulome?

We have previously shown that the hematopoietic developmental TF MYB is controlled by both SUMO conjugation and SUMO binding (21, 22, 23, 24, 25) and analyzed in detail the chromatin occupancy and target genes of WT MYB (45). However, how its SUMO status, both regarding SUMO conjugation and SUMO binding, affects the gene programs it controls remains to be investigated. A basic question is whether the level of SUMOylation of MYB alters its profile of target genes in a qualitative sense, affecting a different set of genes, or whether the effect is mainly quantitative affecting the activity of MYB and thereby the expression levels of the same set of target genes.

In order to investigate which set of target genes are affected by the SUMO status of MYB, we expanded our previous transcriptome profiling by sequencing the transcriptome of K562 cells after rescue of endogenous MYB knockdown by the SUMO conjugation–deficient 2KR mutant of MYB (2KR-MYB) as well as by the SUMO-binding deficient SIM mutant of MYB (ANAA-MYB) (for details on our rescue strategy, see Experimental procedures and Fig. S1*A*).

**Figure 1:**
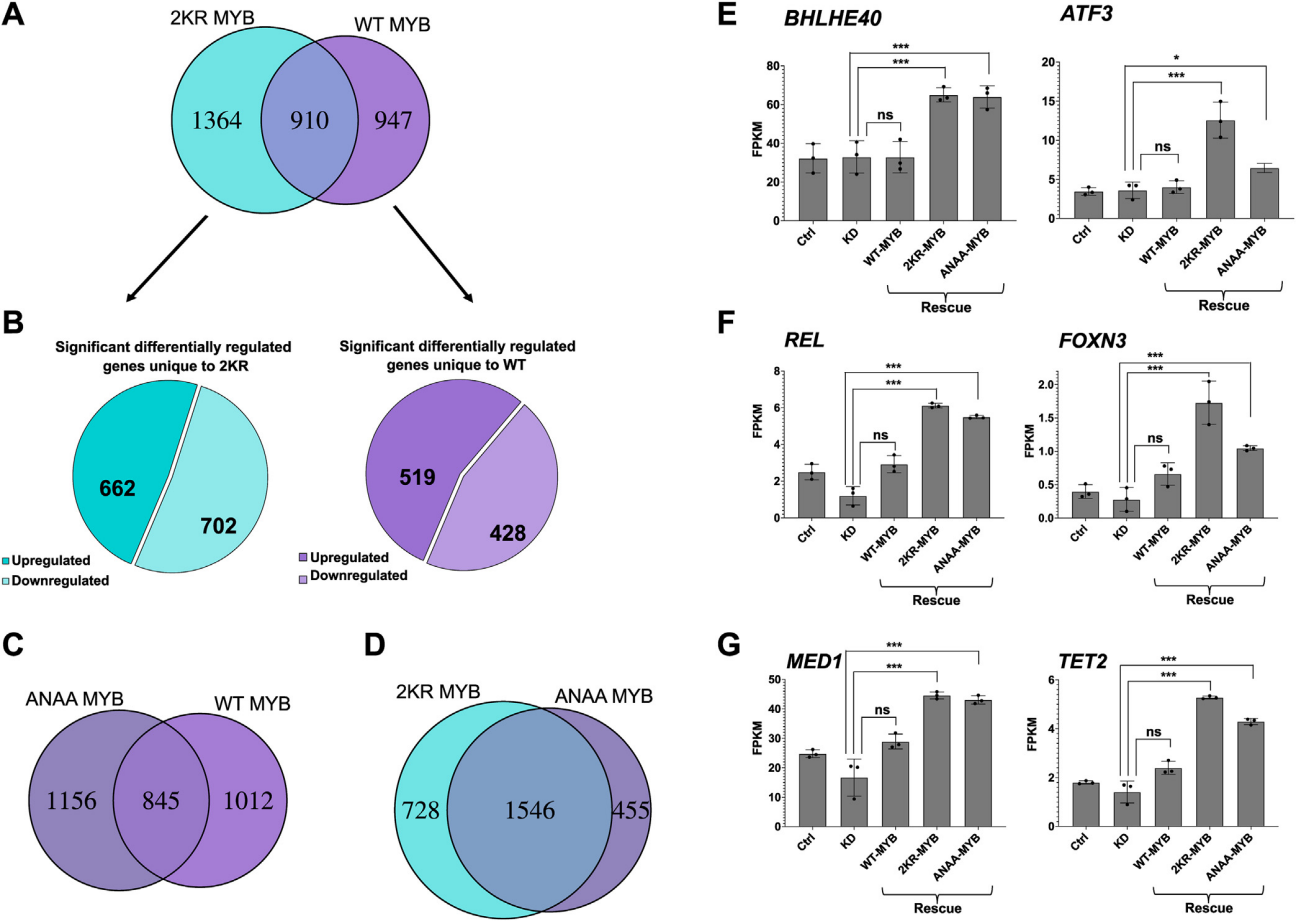
**Significantly differentially regulated genes between rescue of endogenous MYB knockdown with SUMO-negative and SUMO-positive versions of MYB.***A*, intersection between 2KR-MYB and WT-MYB (n = 910). The Venn diagram shows the number of significantly differentially regulated genes that are unique to 2KR (n = 1364) and unique to WT-MYB (n = 947) as determined by the log2 FC (fold change) q-value cutoff of 0.01. The q-value is a Benjamini and Hochberg (BH) adjusted *p*-value to control for FDR (false discovery rate). *B*, pie charts showing proportion of upregulated and downregulated genes. *Left panel* shows the proportion of upregulated genes (n = 662) and downregulated genes (n = 702) that are unique to 2KR-MYB (n = 1364). *Right panel* shows the proportion of significantly differentially upregulated genes (n = 519) and downregulated genes (n = 428) unique to WT-MYB compared to 2KR-MYB (n = 947). *C*, intersection between ANAA-MYB and WT-MYB (n = 845). The Venn diagram shows significantly differentially regulated genes unique to ANAA-MYB (n = 1156) and the corresponding WT list (n = 1012). *D*, intersection between 2KR-MYB and ANAA-MYB (n = 1546). The list of significantly differentially regulated genes from the Venn diagrams belonging to the different categories displayed in (*A*–*D*) are provided as Table S2. *E* and *F*, examples of expression profiles of TFs among the enhanced gene groups upon 2KR-MYB rescue (n = 662, Fig. 6*B*). The expression profiles of *BHLHE40*, *ATF3*, *REL*, and *FOXN3* are shown across ctrl, MYB KD, and rescue with various MYB versions *G*, examples of expression profiles of transcriptional coactivators (MED1) and epigenetic modifiers (TET2) among the enhanced gene groups upon 2KR-MYB rescue (n = 662, Fig. 6*B*). The expression profiles of *MED1* and *TET2* are shown across ctrl, MYB KD, and rescue with various MYB versions. To illustrate the rescue for individual genes, we extracted data from the RNA-Seq FPKM values for each replicate to estimate mean ± SD as shown. Significance was evaluated as in the global analysis by looking at the BH-adjusted *p*-value (q-value) <0.01 on selected pairs and indicated with brackets according to BH-adjusted *p* value (∗*p* < 0.01; ∗∗∗ *p* < 0.0001; ns *p* > 0.01). KD, knockdown; SUMO, small ubiquitin-like modifier protein; TF, transcription factor.

**Figure 2:**
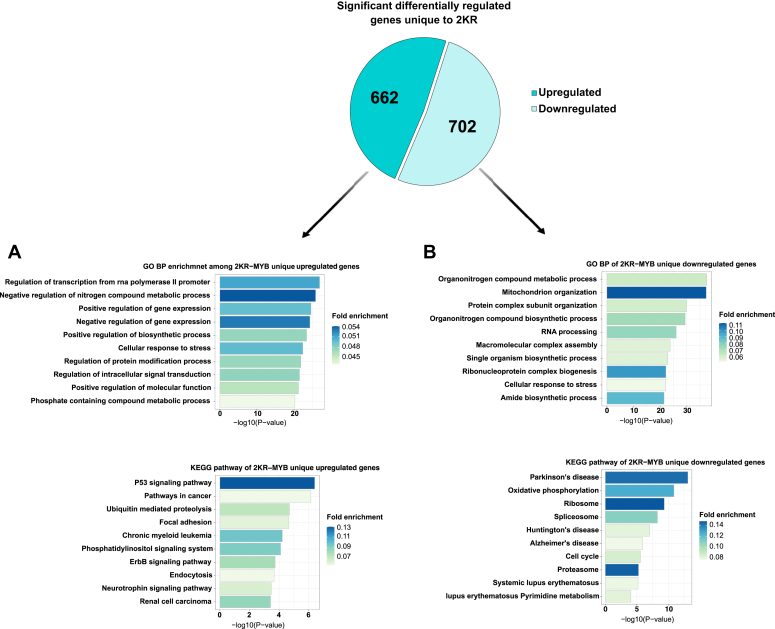
**Gene set enrichment analysis for the significantly differentially regulated genes unique to 2KR-MYB with respect to WT-MYB (n = 1364).***A*, GO terms of biological processes and pathway enrichments for significantly upregulated genes (n = 662). *B*, GO terms of biological processes and pathways enrichment for significantly downregulated genes (n = 702). The GSEA of biological processes as well as KEGG pathway analyses were made using MSigDB database (v6.1) (98). Those GO terms and pathway enrichments with Benjamini and Hochberg adjusted *p* values ≤0.05 were used in the analysis, and the top ten terms were displayed. GO, gene ontology; GSEA, gene set enrichment analysis.

When we explored the differential expression profiles of these rescues, we observed a large group of differentially expressed genes showing both qualitative and quantitative changes in the MYB regulome (Figs. 1 and 2). For the overlap comparisons, we took into consideration all the significantly differentially regulated genes with respect to the endogenous MYB knockdown set and identified a total of 1857, 2274, and 2001 differentially regulated genes within the WT-MYB rescue, 2KR-MYB rescue, and ANAA-MYB rescue sets, respectively.

We first focused on the effect of removing SUMO conjugation from MYB by comparing the rescue by 2KR-MYB *versus* WT-MYB. A large number of genes were found to be nonoverlapping in these two groups (Fig. 1*A*). Of the 2274 differentially regulated genes in the 2KR-MYB rescue set, 1364 were unique to this gene set compared to WT-MYB rescue, while 910 genes were shared between the two conditions (Fig. 1*A*), given the statistical cut-off used. This suggests that the SUMO-negative 2KR mutant of MYB largely induces a qualitatively different gene program than WT MYB. We performed a gene set enrichment analysis (GSEA) of the uniquely differentially regulated genes of the 2KR mutant of MYB to get an idea of what this qualitative difference implied. The significantly upregulated set unique to 2KR (n = 662, Fig. 1*B* left panel) showed top significant enrichment for regulation of key processes such as transcription and positive and negative regulation of gene expression, as well as protein modification and signaling processes (Fig. 2*A*). Inspection of the specific genes in this enhanced group (n = 662) showed that loss of SUMO conjugation led to upregulation of many genes involved in the regulation of transcription, epigenetic modifications, posttranslational modification processes, and signaling, suggesting that the SUMO status of MYB regulates its developmental control of these key processes. As examples, we observed target genes encoding key TFs such as *BHLHE40, ATF3, REL*, and *FOXN3* (as well as *BACH1, FOXO3, JUN, NF1, SMAD3, STAT5B, STAT6*, and more), genes encoding transcriptional coactivators such as the mediator subunit *MED1*, epigenetic modifiers such as *TET2* (as well as *TET3*, *KDM6A KDM6B, KDM4A, KDM5A*, and *KDM5C*), as well as the kinase HIPK1, all being upregulated by the SUMO-negative 2KR-MYB mutant (selected example expression profiles are shown in Fig. 1, *E*–*G*). Furthermore, in the shared group (n = 910), additional genes in the same category were more upregulated in the 2KR-MYB rescue relative to the WT-MYB rescue. These included the histone acetyl transferases *KAT6A* (*MOZ/MYST3*) and *CREBBP* (*CBP/KAT3A*), as well as the kinase *HIPK2*. It is also noteworthy that we observed enrichment of several biological pathways related to cancer and diverse signaling pathways in our GSEA of the upregulated genes unique to 2KR-MYB (Fig. 2*A*). Note that the size of the group we have defined as unique to 2KR-MYB rescue compared to the group shared between 2KR-MYB and WT-MYB rescue (*i.e.*, qualitative *versus* quantitative changes) depends on the statistical cut-off, which we have set rather stringent to q <0.01. This is illustrated in the examples shown in Figure 1, *E*–*G*. While *BHLHE40* and *ATF3* clearly belong to the group unique to 2KR-MYB, the other four genes in the same group would have been in the shared group if we had used a less stringent statistical cut-off.

When we did the same analysis, but now of the effect of removing SUMO binding from MYB by comparing rescue of ANAA-MYB with WT-MYB, we found again a large number of genes being nonoverlapping between the ANAA-MYB and WT-MYB (Fig. 1*C*). Of the 2001 differentially regulated genes in the ANAA-MYB rescue set, 1156 were unique to this gene set compared to the WT-MYB rescue (Fig. 1*C*). Inspection of the genes in this group showed that loss of SUMO binding led to upregulation of many of the same genes as observed in the 2KR-MYB rescue set. All the examples of further activated target genes shown in Figure 1, *E*–*G* were also upregulated by ANAA-MYB. In fact, this unexpected similarity was evident from a comparison of the profiles from the rescue with the two SUMO mutants. We know that the SUMO-binding deficient ANAA-MYB is quite distinct from 2KR-MYB with regard to association with SUMO and molecular alteration (25). Yet, when we investigated the overlap of differentially regulated genes by the two SUMO-deficient rescues (2KR-MYB and ANAA-MYB), we observed a particularly large overlap (1546) (Fig. 1*D*). The mechanistic explanation for this similarity is not obvious but it may be related to group SUMOylation (1, 18, 19), where networks of SUMO conjugation and SUMO binding contribute to the same overall effect.

When the SUMOylation balance is changed, one may expect many indirect effects on the global gene expression profile. A relevant question is therefore how many of the alterations in the 2KR- and ANAA-MYB transcriptomes are direct. The precise answer to this question would require separate chromatin immunoprecipitation (ChIP)-Sequencing (Seq) analyses for each mutant, which we do not have. However, we have recently analyzed extensively the chromatin occupancy of WT-MYB in K562 cells (45). We decided to use this as a proxy for the occupancy of the mutants, well aware of the limitations of such an approach. Among the several TFs whose SUMO conjugations has been previously reported to affect their DNA-binding property, inhibitory effects seem to be caused by SUMOylation within or close to the DNA-binding domain (DBD) or due to indirect effects on posttranslational modifications or interaction partners, none of which seems relevant for MYB. Although the SUMO-conjugation sites in MYB are far from its DBD, we cannot fully exclude indirect effects since mSOX2 and FOS (46, 47) exhibit examples where SUMO-conjugation sites outside of their DBDs still affect DNA-binding properties through unknown mechanisms.

**Figure 3:**
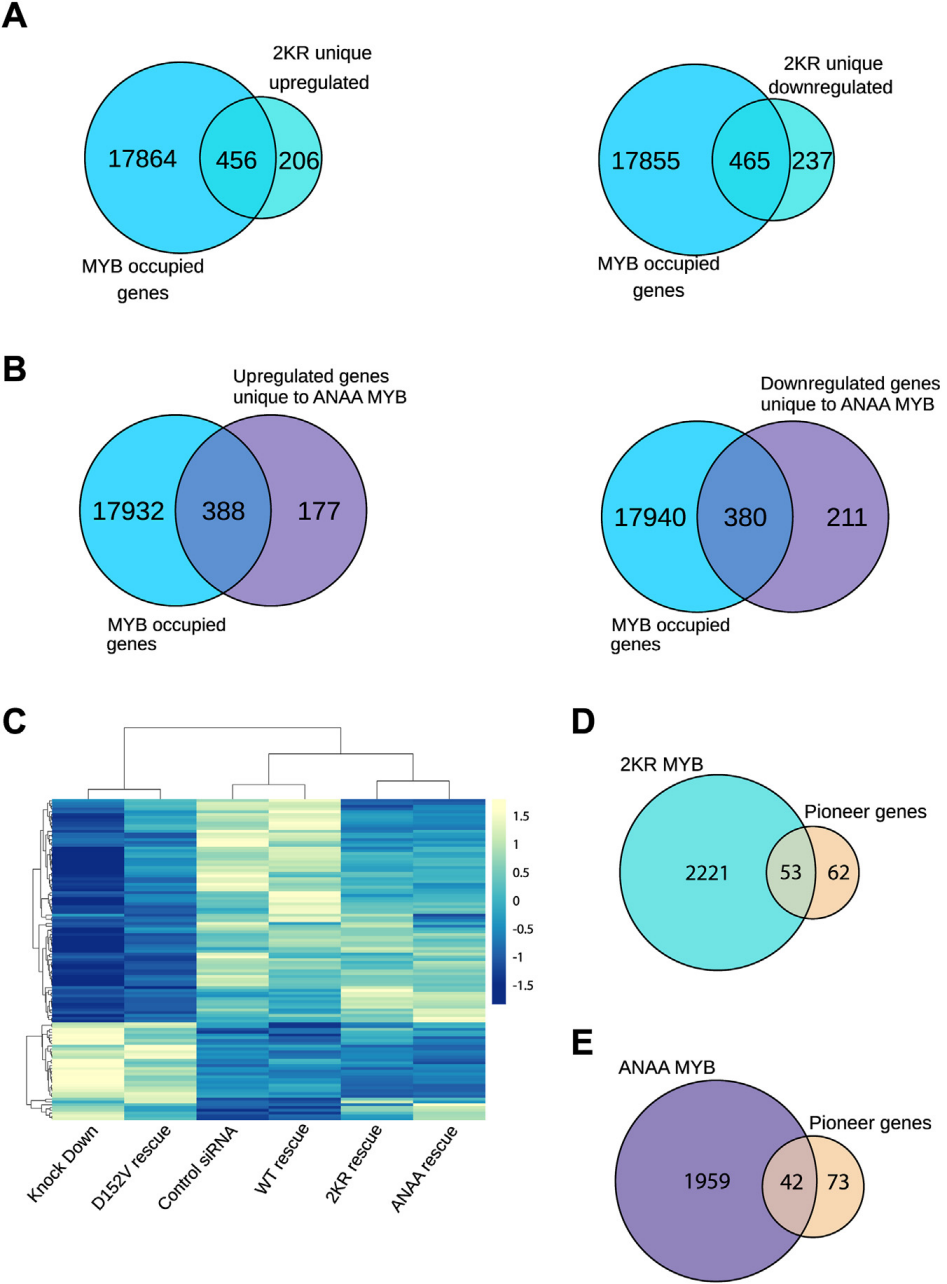
**Direct target genes of SUMO-deficient MYB.***A*, direct target genes of 2KR-MYB. The Venn diagram in the *left panel* shows overlap (n = 456) between significantly upregulated genes by 2KR-MYB (n = 662, Fig. 1*B*) and MYB occupied genes from (45). The *right panel* shows similar overlap but for the significantly downregulated genes in 2KR-MYB (n = 702, Fig. 1*B*). *B*, direct target genes of ANAA-MYB. The Venn diagram in the *left panel* shows overlap (n = 388) between significantly upregulated genes by ANAA-MYB and MYB occupied genes from (45). The *right panel* shows similar overlap but for the significantly downregulated genes in ANAA-MYB. For these overlap analyses, we expanded the list of MYB occupied genes reported in Lemma and Ledsaak *et al*. (45), where in addition to linking MYB peaks with STITCHIT-derived REMs, we incorporated a distance-based annotation to associate MYB peaks to the nearest TSS as described in Lemma *et al.* (100). *C*, a fraction of MYB’s pioneer genes is sensitive to MYB’s SUMO status. Hierarchical clustering of the pioneer genes is (n = 115 (45)). The cluster heat map was generated using ClustVis webtool (123). Each row represents a gene. *D*, overlap between significantly differentially regulated genes in 2KR-MYB and the pioneer genes. *E*, overlap between significantly differentially regulated genes in ANAA-MYB and pioneer genes. SUMO, small ubiquitin-like modifier protein; TSS, transcriptional start site.

With this reservation, we performed the analysis assuming that the DNA-binding properties of the 2KR and ANAA mutations are not largely altered and that the ChIP-Seq profile of WT-MYB still reflects to a reasonable degree the genome-wide chromatin occupancy of the mutants. Specifically, we asked what fraction of the gene set becoming uniquely activated by 2KR-MYB (n = 662) also has enrichment of MYB ChIP-Seq signal. As shown in Figure 3*A*, left panel, we observed 456 genes with MYB occupancy at their loci among the uniquely upregulated genes in 2KR-MYB (n = 662). The same analysis for the downregulated gene set (n = 702, Fig. 1*B*, left panel) showed 465 genes with MYB occupancy at their loci (Fig. 3*A*, right panel). This suggests that more than 50% of genes either activated or repressed by 2KR-MYB could be direct target genes of MYB and their regulation being dependent on the SUMO conjugation status of MYB.

We did the same analysis for the gene set becoming uniquely differentially regulated by ANAA-MYB (n = 1156, Fig. 1*C*), after splitting them into those that are activated by ANAA-MYB (n = 565) and those that are downregulated by ANAA-MYB (n = 591). A total of 388 genes seemed to be occupied by MYB (Fig. 3*B*, Left) from the upregulated set and 380 genes from the downregulated set (Fig. 3*B*, Right). This indicated that the activity of more than 50% of genes either activated or downregulated by ANAA-MYB could be direct MYB target genes and their regulation being dependent on the SUMO-binding status of MYB. MYB occupancy at the loci of selected target genes affected by the SUMO status of MYB listed earlier and/or highlighted in Figure 1, *E*–*G* are shown in Fig. S3.

While a high fraction of genes affected by MYB knockout and rescue can be linked to an associated MYB peak (45) and thus be classified as direct targets, the opposite question can also be addressed: how large portion of all MYB-binding sites do have an association with genes affected by MYB knockout and rescue? In general, not all TF-binding events are necessarily functional in the sense of changing the expression of associated genes. Often, a relatively small overlap has been observed between TF occupancy and the expression of neighboring genes, in the order of 10 to 25% in higher eukaryotes (48) or less (49). For MYB, we have estimated that only 4% was found to significantly change expression upon MYB knockdown (45). Still, the question of functionality may be more complex for a pioneer factor like MYB (42) since a binding event may also cause changes in chromatin states that indirectly affect the subsequent expression patterns in a differentiation process. In addition, some genes may be modestly affected but not enough to pass the stringent threshold we have set to define target genes.

We have recently shown that MYB is a pioneer TF and a single amino acid mutation (D152V) of MYB abrogates its pioneering property (42). We have defined genes that were rescued by WT-MYB but not rescued by the D152V mutant as pioneer target genes (42). We next asked whether this subgroup of pioneer target genes might be affected differently by the SUMO status of MYB compared to the nonpioneer group of targets. Hierarchical clustering of the pioneer genes (n = 115) (Fig. 3*C*) revealed that the expression profile of the pioneer genes in the rescue sets by the SUMO mutants (2KR-MYB and ANAA-MYB) generally resembles that of the control and WT sets and clusters into one large group together with the control and WT sets (Fig. 3*C*). Within this large cluster group, the expression profile of the control and WT sets closely resemble each other, whereas the expression profiles of 2KR-MYB and ANAA-MYB sets closely resemble each other forming two subgroups. When we analyzed how many of these pioneer genes were rescued by the SUMO conjugation or SUMO-binding deficient MYB, the expression profiles of 53 out of 115 genes were rescued by the 2KR-MYB (Fig. 3*D*) and 42 were rescued by ANAA-MYB (Fig. 3*E*), suggesting that a fraction of the pioneer genes are not affected by the SUMO status of MYB, whereas a slightly bigger fraction of pioneer genes are affected. List of the pioneer genes that appear to be affected by the SUMO status of MYB are provided in Table S1.

### SENP1 is a MYB target gene

In the analysis above, we have studied MYB with altered SUMO status caused by mutations. It is less clear how such changes may play a role in a normal situation. One obvious way of changing the level of SUMO conjugation is by altering the balance between SUMO-conjugation enzymes and SUMO-deconjugation enzymes. Our previous studies had shown that the TF MYB can be activated by SENP1 in transfection assays (24, 25). Although SENP1 is expressed in many tissues, it appears to play a key role at specific stages of hematopoiesis (50, 51). Therefore, we examined whether MYB, a fundamental TF in hematopoiesis, might regulate the expression of *SENP1*.

**Figure 4:**
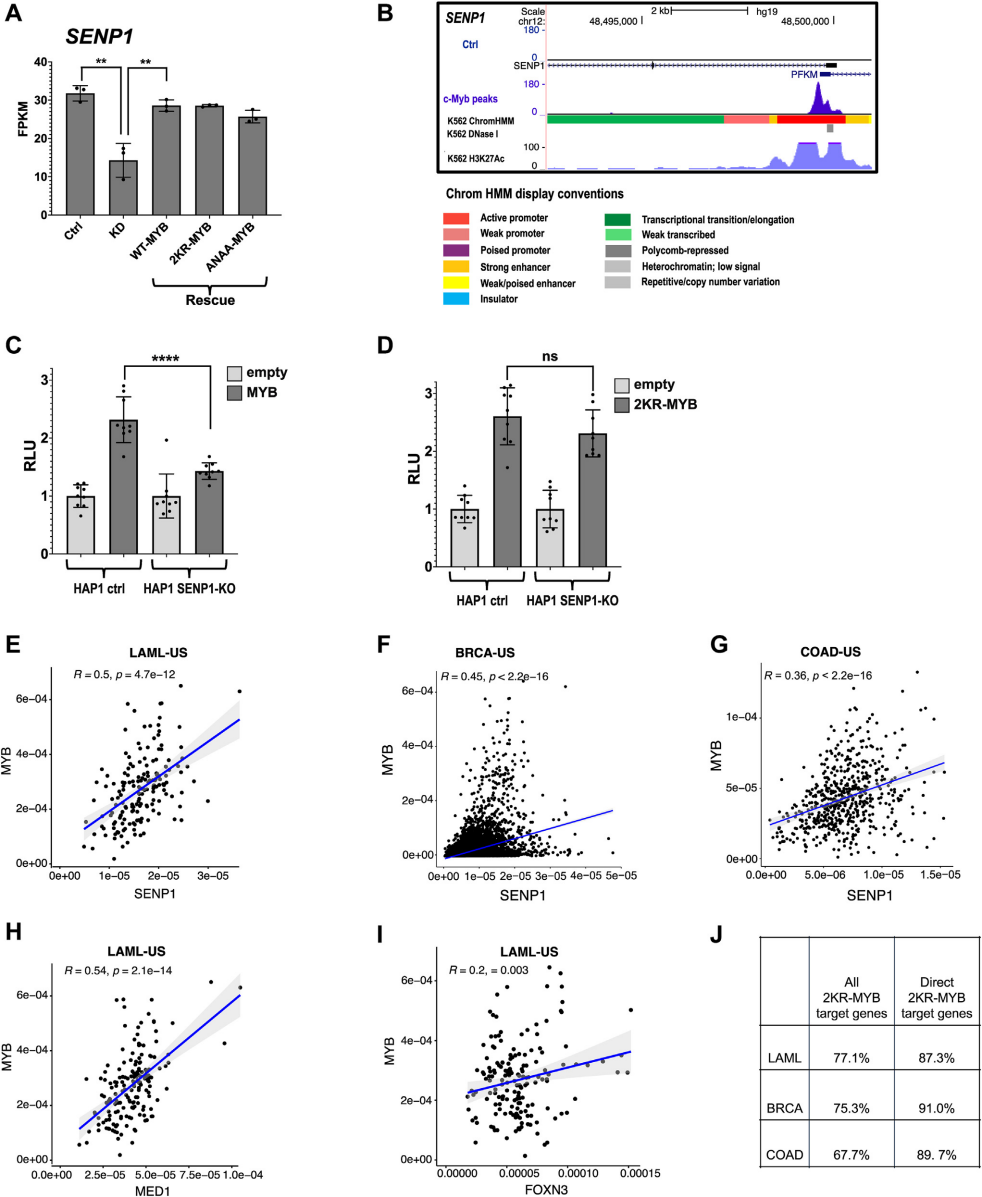
**MYB is a direct regulator of SENP1 creating an autoactivation loop.***A*, MYB positively regulates the expression of SENP1. The expression profile of SENP1 in K562 cells stably integrating WT-MYB and SUMO MYB mutations are shown. K562 cells were transfected with control siRNA and MYB siRNA (siU2992, targeting the 3′-UTR of MYB), and KD of endogenous MYB was rescued by WT, 2KR, and ANAA versions of MYB. To illustrate the rescue for *SENP1*, we extracted data corresponding to SENP1 from the RNA-Seq FPKM values for each replicate to estimate mean ± SD as shown. *B*, sharp TF peak showing MYB-ChIP-Seq occupancy at the TSS of the *SENP1* locus. Visualization of the track was made using the UCSC genome browser (108). Differential gene expression was calculated using *cuffdiff* in the cufflinks suite version 2.2.1. *C*, a test of MYB’s autoactivation loop. Luciferase reporter assay in HAP1 control and SENP1-KO cell lines in the presence or absence of MYB. *D*, similar setup as “(*E*)” where MYB was replaced with 2KR-MYB. HAP1 control and SENP1-KO cells were transfected with 0.2 μg/μl of either 3×FLAG-MYB or 3×FLAG-2KR-MYB plasmids and 0.1 μg/μl of the MYB-responsive reporter plasmid (pGL4b-3×MRE(GG)-MYC-aab3). The reporter assay results are presented as mean ± SD of independent biological replicates, each performed atleast in triplicates (n ≥ 3). Significance for (*A*) and the reporter assays was evaluated by unpaired, two-tailed *t*-tests on selected pairs and indicated with *p* values (∗∗*p* < 0.01; ∗∗∗∗*p* < 0.0001; ns *p* > 0.05). *E*–*G*, expression of MYB shows positive correlation with expression of SENP1 in leukemia, breast, and colorectal cancer patients. Pearson correlations are computed between MYB and SENP1 expressions in LAML, BRCA, and COAD cohorts from TCGA. The scatterplots compare the expression of SENP1 (x-axis) and the expression of MYB (y-axis). The *blue lines* represent the fitted Pearson linear correlation with the *gray* zone representing the 95% confidence interval (Pearson R coefficients and associated *p* values are provided in the *top-left* corner). *H* and *I*, example Pearson correlations of selected 2KR-target genes (Fig. 1*B*, n = 662). Expression of MYB shows positive correlation with expression of MED1 and FOXN3 in leukemia cancer patients. Pearson correlation is computed between MYB and selected target expressions in LAML cohorts from TCGA. Details of statistical analysis are similar to (*E*–*G*). *J*, Pearson correlation coefficients between MYB expression and 2KR-MYB target genes among the TCGA, LAML, BRCA, and COAD cancer cohorts. The correlation coefficients are computed for both direct target genes of MYB in 2KR set (Fig. 3*A*) and all 2KR-MYB target genes (Fig. 1*B*). MRE, MYB responsive element; SENP, sentrin-specific protease; SUMO, small ubiquitin-like modifier protein; TCGA, The Cancer Genome Atlas; TF, transcription factor; TSS, transcriptional start site.

When we examined our transcriptomic data (42), along with the current RNA-Seq data for rescue of endogenous MYB knockdown with SUMO mutants of MYB, we found that MYB in fact regulates the expression of SENP1. We observed a significant decrease of SENP1 mRNA expression level when endogenous MYB was knocked down (Fig. 4*A*). This endogenous MYB knockdown of SENP1 was rescued by both the WT- and SUMO-negative (2KR- and ANAA-) MYB. Finally, we examined whether SENP1 is a direct target gene of MYB by analyzing the occupancy of MYB by ChIP-Seq from our recent data (45). As shown in Figure 4*B*, there is a sharp MYB ChIP-Seq peak at the transcriptional start site (TSS) of the *SENP1* loci, supporting our hypothesis that SENP1 is a direct target of MYB.

We found it intriguing that a TF, which is itself controlled by SUMO conjugation and SUMO binding (23, 24, 25), controls the levels of a key player in the SUMO system, such as SENP1. By affecting the balance between SUMO modification and demodification, MYB may have far-reaching effects. The implication is that MYB is able not only to control a specific gene program directly but also may affect the SUMO landscape indirectly through SENP1 regulation.

### An autoactivation loop for MYB

Since MYB itself is repressed by SUMOylation (21, 22, 23) and activated by SENP1 (24), MYB-dependent regulation of *SENP1* would be expected to cause activation of MYB itself creating an autoactivation loop. The implication would be that the activity of MYB would increase in a nonlinear fashion with its protein level because increasing levels of SENP1 would contribute to lower levels of repressive SUMO conjugation and thus a more active form of MYB. Another implication would be a reduced activity of MYB in the absence of SENP1. This is what would be expected if the activity of MYB is dependent on the normal endogenous level of SENP1. Moreover, no such reduction should be observed for the SUMO conjugation–negative 2KR mutant of MYB. We tested this hypothesis by performing a reporter assay using HAP1 cells lacking SENP1, that is, a SENP1-KO cell line and with the parental cell line as control. The HAP1 cells are derived from a near-haploid human cell line (52), in which the *SENP1* gene was inactivated by a CRISPR-Cas9–generated frameshift mutation into the coding sequence of *SENP1*. We transfected the cells with a reporter plasmid consisting of three MYB responsive elements (3×MRE) and an effector plasmid encoding 3×FLAG-MYB. This allowed us to compare directly the MYB activity in the presence and absence of endogenous SENP1 as well as the importance of MYB being SUMO-conjugated (Fig. 4, *C* and *D*). Here the reporter activity of MYB was significantly lower in the SENP1-KO cell line than the WT control (Fig. 4, *C* and *D*). Moreover, the 2KR mutant version of MYB, not modulated by SUMOylation, showed comparable activity in the two cell lines, being unaffected by the loss of SENP1 (Fig. 4*D*). These observations are consistent with the autoactivation hypothesis.

Since MYB is overexpressed in many cancers including leukemia, breast, and colorectal cancers (27, 53, 54, 55, 56, 57), it may under these conditions be less SUMO-repressed according to the autoactivation hypothesis due to the deregulation of SENP1 expression. If so, overexpressed MYB would be expected to approach a 2KR version of MYB. We therefore asked whether overexpressed *MYB* correlates with *SENP1* expression in leukemia, breast, and colorectal cancer patients. For this, we took advantage of RNA-Seq data for LAML (Acute Myeloid Leukemia), BRCA (Breast Invasive Carcinoma), and COAD (Colon Adenocarcinoma) cohorts, respectively, from The Cancer Genome Atlas (TCGA) (58). When Pearson correlations between *MYB* and *SENP1* expressions were computed from these cohorts, we observed a significant positive correlation, with Pearson correlation coefficient R = 0.5, *p*-value = 4.7e^−12^; R = 0.45, *p*-value <2.2e^−16^; and R = 0.36, *p*-value <2.2e^−16^ in LAML, BRCA, and COAD, respectively (Fig. 4, *E*–*G*). As a consequence, we expected not only higher levels of the MYB protein but also a more active MYB protein because of altered SUMOylation balance.

### Target genes affected by SUMO status of MYB analyzed in cancer cohorts

We expanded this analysis and used the same TCGA approach to evaluate the broader picture of target genes found to be affected by the SUMO status of MYB in our model cell line K562, well aware of the many perturbations that may influence the expression profiles in cancer patients. According to the autoactivation hypothesis, overexpressed MYB should behave closer to 2KR-MYB due to its effect on SENP1 expression. We therefore asked whether overexpressed MYB correlates in the cancer patient cohorts with higher expression of targets enhanced by 2KR-MYB in K562 similar to the analysis we did with SENP1 expression above. Therefore, we analyzed all target genes uniquely regulated by 2KR-MYB (Fig. 1*B*, left panel). Among these 2KR-MYB target genes being also expressed in the respective cancer cohorts, 77.1%, 75.3%, and 67.7% showed a significant correlation between their expression and the expression of MYB in the LAML, BRCA, and COAD cancer cohorts, respectively (Fig. 4*J*). A couple of examples from the LAML cohort are shown in Figure 4, H and I, and the full list with statistics is shown in Table S3. When we compared the correlations between MYB expression and its targets in the 2KR-MYB set that are also defined as direct 2KR-MYB targets (Fig. 3*A*), we observed even stronger correlations, 87.3%, 91.0%, and 89.7% in the LAML, BRCA, and COAD cancer cohorts, respectively (Fig. 4*J*). The full list of correlations for direct 2KR-MYB targets with statistics is shown in Table S4. Generally, we observed a slightly better correlation between MYB expression and upregulated targets in the 2KR-MYB set compared to the downregulated targets. The overall high frequency of positive correlations observed is consistent with the patterns found in K562 and with the autoactivation hypothesis.

### SENP1 interacts with UXT

**Figure 5:**
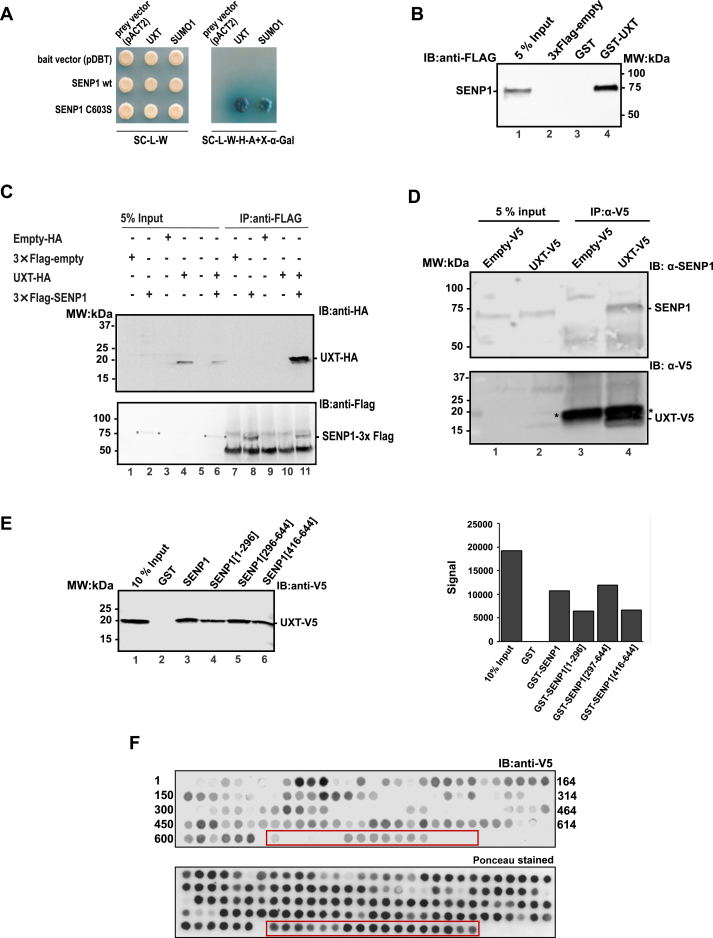
**UXT interacts with SENP1.***A*, identification of UXT as interaction partner of SENP1 by Y2H screen and validation of selected positive cDNAs by remating in the pACT2 vector (UXT and SUMO1), crossed with the indicated bait plasmids (in the pDBT vector). The *left panel* shows growth on the control plate (SC/-trp/-leu medium) that selects for diploid a/α-cells containing both pDBT and pACT2 plasmids. The *right panel* shows growth on SC/-trp/-leu/-his/-ade/+X-α-Gal medium, where growth and color is dependent on interaction. We used catalytic dead mutant of SENP1 (SENP1 C603) because SENP1 is toxic to the yeast cells. *B*, GST pull-down–binding assay was done with GST-UXT and lysate from COS-1 cells transfected with 3×FLAG-SENP1. Twenty four hours after transfection, the COS-1 cells were lysed in lysis buffer and incubated with GST-fused UXT that was bound to glutathione beads. The bound protein was separated by SDS-PAGE, and the immunoblot was analyzed using anti-FLAG antibody (1:1000) and HRP-linked anti-mouse secondary antibody (1:10,000). Five percentage of the total cell extract used in pull-down was loaded as control. *C*, co-immunoprecipitation of UXT with SENP1. COS-1 cells were transfected with the indicated combinations of pCIneo-3×FlAG-SENP1 and pDEST-HA-UXT plasmids. COS-1 cells were lysed, immunoprecipitated with FLAG beads, separated by SDS-PAGE, and UXT was revealed by immunoblotting using anti-HA antibody. Five percentage of the total transfected cell lysates were loaded as input references. *D*, co-immunoprecipitation at endogenous level of SENP1 with UXT-V5. The K562 nuclear extract from UXT-V5 (clone C5) was incubated with Protein G magnetic beads coupled to 2 μg anti-V5 monoclonal antibody. A nuclear extract from an empty-V5 stable K562 cell line was used as a control. *E*, interaction mapping using GST pull-down–binding assay (*left panel*) and quantitative western analysis of the immunoblot (*right panel*). GST pull-down–binding assay was done with different deletion constructs of GST-bound SENP1 and lysate from COS-1 cells transfected with UXT-V5. Twenty four hours after transfection, the COS-1 cells were lysed in lysis buffer and incubated with GST-fused SENP1 deletion constructs that were bound to glutathione beads. The bound proteins were separated by SDS-PAGE, and the immunoblot was analyzed using anti-V5 antibody (1:5000) and IRDye 680 RD (LI-COR) anti-mouse secondary antibody (1:10,000). *F*, interaction mapping using peptide arrays. SENP1 peptide arrays of 20 amino acids long were spotted in a nitrocellulose membrane with a sliding step of five amino acids. The array was incubated with 10 ng of purified GST-bound UXT-V5 in lysis buffer. Bound UXT was revealed with anti-V5 antibody and IRDye 800 CW (LI COR) anti-mouse secondary antibody (1:10,000) (*upper panel*). *Lower panel* shows the same peptide array with ponceau staining. In the figure, extra peptides that are not relevant for this study and were spotted at the end after the SENP1 peptides are displayed with a *red rectangle* surrounding them. SENP, sentrin-specific protease; SUMO, small ubiquitin-like modifier protein; UXT, ubiquitously expressed transcript; Y2H, yeast two-hybrid.

In our previous studies, we showed that SENP1 activated the TF MYB (24, 25), and based on our observation above that SENP1 is a target gene of MYB, we went one step further and started to map the interactome of SENP1 to better understand its role as an activator of transcription in general and of MYB in particular (15). In a yeast two-hybrid (Y2H) screen utilizing a human thymus complementary DNA (cDNA) library, as described in (15), *UXT* was among the cDNAs that gave the strongest blue color on selective X-gal plates (Fig. 5*A*). UXT, also named ART27 (Androgen Receptor Trapped clone-27), is a small (∼18 kDa) prefoldin-like protein (59, 60, 61, 62, 63, 64, 65, 66). There are two variants expressed from the *UXT* gene in humans, a longer isoform (169 aa long) and a shorter isoform (157 aa long), as a result of an in-frame downstream start codon (62). The two variants differ in their subcellular localization, with the longer variant found exclusively in the cytoplasm, whereas the shorter variant, isolated in this Y2H screen, is almost exclusively localized in the nucleus (62).

In order to validate the UXT–SENP1 interaction, we carried out pull-down assays and co-immunoprecipitation (co-IP) experiments, which both supported that UXT and SENP1 are physically interacting. Full-length FLAG-tagged SENP1 was efficiently pulled down from a COS-cell extract with glutathione S-transferase (GST)-UXT, but not with GST alone (Fig. 5*B*). Similarly, using lysates from COS-1 cells transfected with 3×FLAG-SENP1 and HA-UXT, we found that UXT was co-immunoprecipitated with SENP1 (immunoprecipitated with anti-FLAG antibody) (Fig. 5*C*). We next validated this interaction under more stringent conditions using semi-endogenous co-IP (Fig. 5*D*). For this, we generated a stable K562 cell line expressing UXT with a C-terminal V5 tag. Using nuclear extracts from this cell line, we observed that endogenous SENP1 was immunoprecipitated with the integrated V5-tagged UXT protein (Fig. 5*D*).

After validating the interaction, we asked which regions in UXT and SENP1 were involved in the interaction. Since UXT is a rather small protein (∼18 kDa) compared to SENP1 (∼75 kDa), we decided to map the interaction on SENP1 in a GST-pulldown assay. Three different deletion constructs of SENP1 along with the full-length protein were used, encoding an N-terminal segment (SENP1[1–296]), a C-terminal segment (SENP1[297–644]), and a minimal catalytic domain (SENP1[416–644]). Surprisingly, UXT bound to all the four constructs, but not to the GST-control (Fig. 5*E* left panel). However, the strongest binding, based on quantitative Western blot (WB) analysis, appears to be the region between residues 297 and 416 (Fig. 5*E* right panel). In an effort of narrowing down the interaction site to a specific region of SENP1, we mapped the interaction of UXT on an SENP1 peptide array. The array consisted of SENP1 peptides spotted on a nitrocellulose membrane, each 20 amino acids long, with a sliding step of five amino acids, which resulted in a 15 amino acids overlap between the preceding and succeeding peptide. After binding of recombinant GST-UXT-V5 and probing with anti-V5 antibody, we confirmed that UXT interacts with both the N-terminal and C-terminal parts of SENP1 (Fig. 5*F*). The sequences in the underlying SENP1 peptides along with UXT-binding information are shown in Fig. S4. A continuous region including some strong spots were observed in the C-terminal part between residues 297 and 644, with the longest continuous binding region being between residues 451 and 644. This is consistent with the observation in the pull-down assay. A similar dual interaction surface on SENP1 was recently reported for its interaction with c-Myc (67).

In order to clarify this complex pattern of interactions, we used a combination of sequence comparison, homology modeling, secondary structure and disorder prediction approaches, and the recent full-length SENP1 protein structure prediction from AlphaFold (68, 69) together with our pull-down and peptide array results. As explained in more detail in Supplementary results, we conclude from these analyses that UXT most likely binds to the structured C-terminal domain of SENP1 through a 3D epitope. The binding to the N-terminal domain (which appears to be intrinsically disordered, based on the various predictions and experimentally solved SENP1 protein structure, see Figs. S4–S7) is probably through a linear epitope. This disordered region spans from residue 1 to 415 of the SENP1 protein (Fig. S6*A*).

### Characterization of UXT

**Figure 6:**
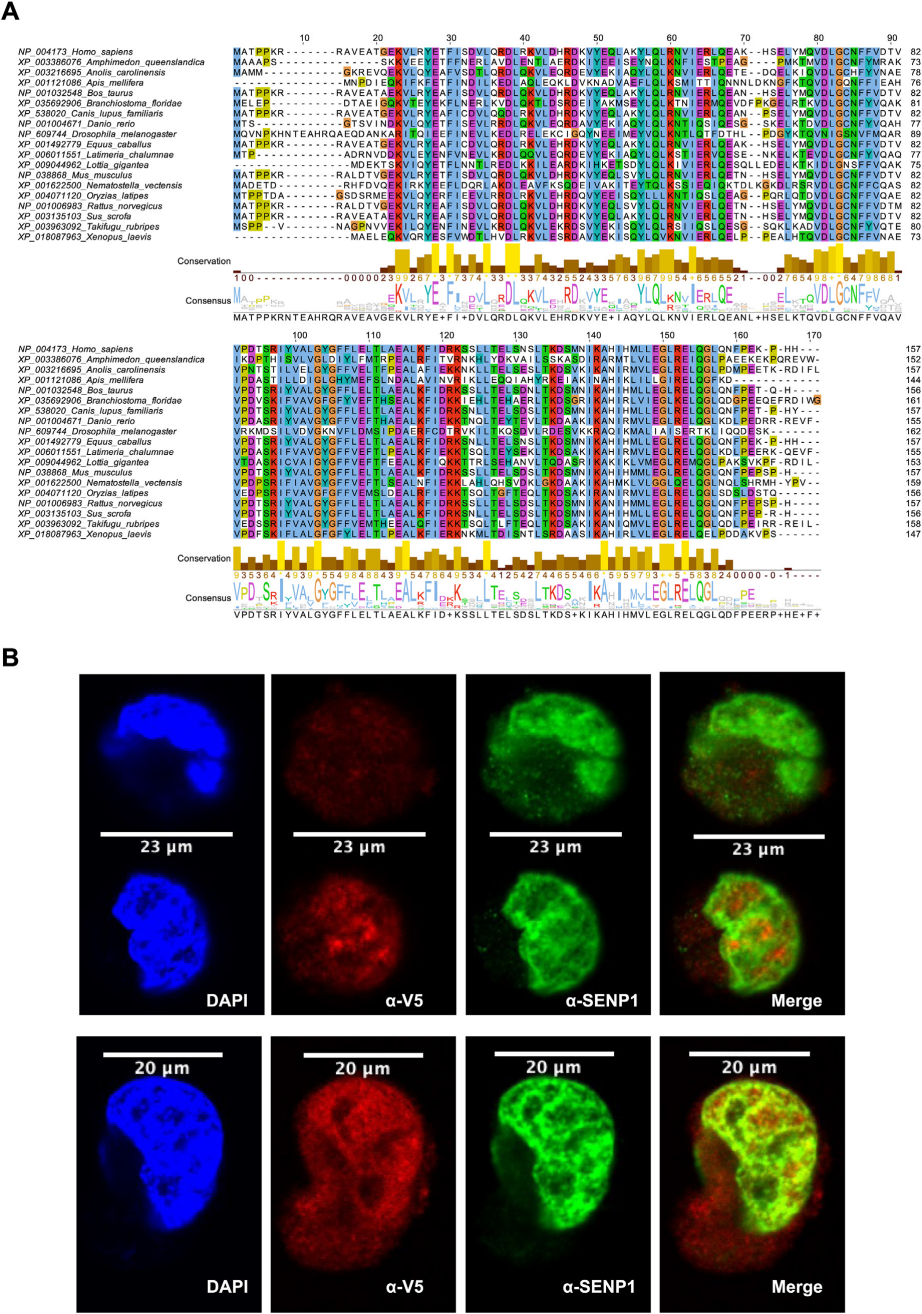
**Characterization of UXT.***A*, multiple sequence alignment (MSA) of homologous sequences of the human UXT protein across various taxa in metazoan. Protein sequences were obtained from NCBI’s Nr. Database using DELTA-BLAST, MSA was generated using the MAFFT algorithm available as web services within Jalview and visualized using Jalview (v.2.10) (109). *B*, UXT and SENP1 were found in the same cellular compartment. Nuclear localization of UXT in K562 cells stably expressing UXT-V5 was observed. The cells were grown on glass slides, fixed with 4% PFA, and probed against mouse anti-V5 monoclonal antibody (1:500) to detect the localization of the stably expressed UXT-V5 and rabbit anti-SENP1 polyclonal antibody (1:500) to detect the localization of endogenous SENP1. Alexa Flour 488 secondary anti-rabbit antibody (1:400) and Alexa Flour 647 secondary anti-mouse antibody (1:100) were used. Nuclear staining was made using DAPI in vectashield mounting medium. Scale bars in μm are indicated on top of each cell. DAPI, 4′,6-diamidino-2-phenylindole; PFA, paraformaldehyde; SENP, sentrin-specific protease; UXT, ubiquitously expressed transcript.

A search for homologous sequences of the human UXT protein using DELTA-BLAST within metazoan organisms revealed that homologous sequences of UXT are found even in basal metazoans such as nematostella and sponge. Multiple sequence alignment of the protein sequences showed that they are quite conserved across several taxa in metazoans (Fig. 6*A*), suggesting that UXT has fundamental functional roles.

To investigate subcellular localization of UXT, we carried out immunofluorescence experiments. Investigation of the subcellular localization of endogenous SENP1 in K562 cells stably expressing low levels of C terminally V5-tagged UXT revealed that SENP1 and UXT were found in the same cellular compartment (Fig. 6*B*). However, the lack of distinct foci precluded an evaluation of precise colocalization. Overall, the nuclear localization of UXT reported here is in line with previous reports (61, 62, 65, 66).

### UXT attenuates the enzymatic activity of SENP1

**Figure 7:**
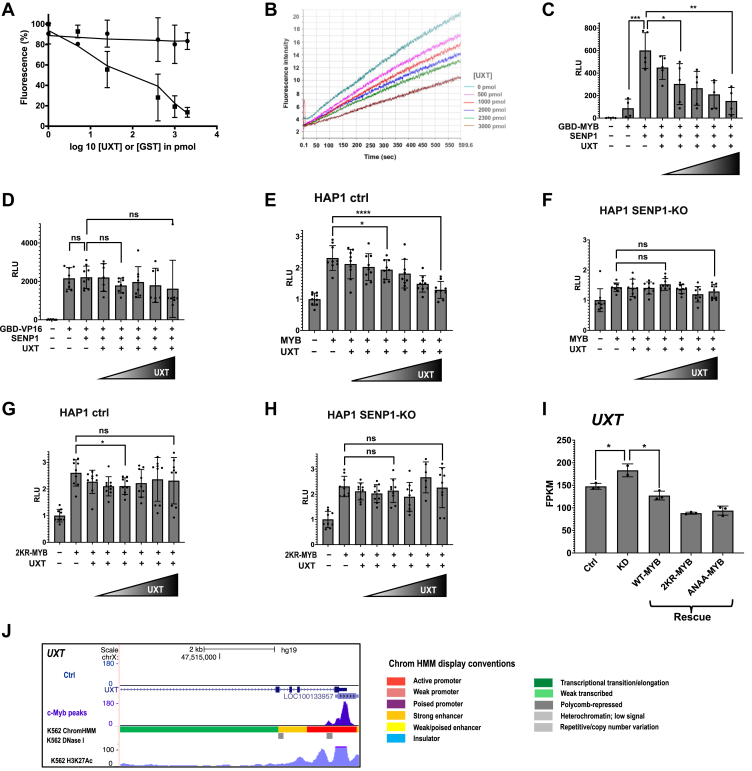
**UXT attenuates the enzymatic activity of SENP1.***A*, *in vitro* de-SUMOylation assay. The effect of increasing amounts of UXT on SENP1’s activity was assayed using a constant amount of SENP1 at 400 pM (picomolar) and increasing amounts of UXT and SUMO1-AMC at 5 μM concentration. Increasing amounts of GST was used as a control. The activity of SENP1 was measured using a fluorometer plate reader. *B*, continuous measurement of SENP1 activity. Similar experimental setup as “(*A*)” where SENP1 at 1000 pM, various concentrations of UXT, and 5 μM SUMO1-AMC were used in the assay. Continuous SENP1 enzymatic activity was measured using Luminescence spectrometer. *C*, luciferase reporter assay. The enzymatic activity of SENP1 in the presence of increasing amounts of UXT was investigated *in vivo* by indirectly measuring the activity of MYB on the luciferase reporter gene. HEK293-C1 cells containing array of Gal4-responsive element stably integrated in front of the luciferase gene were transfected with 0.2 μg/μl MYB[194–640], 0.1 μg/μl 3×FLAG-SENP1, and increasing amounts of UXT-V5 (0.05 μg/μl, 0.1 μg/μl, 0.2 μg/μl, 0.25 μg/μl, and 0.3 μg/μl). *D*, control transfections of the same cells as in (*C*), where MYB was replaced with VP16. *E*, luciferase reporter assay in HAP1 control cell line. The enzymatic activity of endogenous SENP1 in the presence of increasing amounts of UXT was indirectly investigated *in vivo* by measuring the activity of MYB on a reporter gene. *F*, luciferase reporter assay in HAP1 SENP1-KO cell line. HAP1 control (WT) and SENP1-KO cells were transfected with 0.2 μg/μl 3×FLAG-MYB, 0.1 μg/μl of the MYB-responsive reporter plasmid (pGL4b-3×MRE(GG)-MYC-aab3), and increasing amounts of pCIneo-UXT-V5 (0.05 μg/μl, 0.1 μg/μl, 0.15 μg/μl, 0.2 μg/μl, 0.25 μg/μl, 0.3 μg/μl). Transfection of these cells with 3×FLAG-2KR-MYB was used as control in (*G* and *H*). *G* and *H*, luciferase reporter assay in HAP1 control (WT) and HAP1 SENP1-KO cell lines transfected with 3×FLAG-2KR-MYB. All results from the reporter assays are presented as mean ± SD of independent biological replicates, each performed atleast in triplicates (n ≥ 3). Significance was evaluated as reported in Figure 4 and indicated with *p* values (∗*p* < 0.05; ∗∗*p* < 0.01; ∗∗∗*p* < 0.001; ∗∗∗∗*p* < 0.0001; ns *p* > 0.05). *I*, MYB negatively regulates the expression of UXT. To illustrate the rescue for *UXT*, we extracted data corresponding to UXT from the RNA-Seq FPKM values for each replicate to estimate mean ± SD as shown. Significance was evaluated as reported in Figure 4. *J*, sharp TF peak showing MYB-ChIP-Seq occupancy at the TSS of *UXT* locus. Visualization of the track was made using the UCSC genome browser (108). MRE, MYB responsive element; SENP, sentrin-specific protease; SUMO, small ubiquitin-like modifier protein; TF, transcription factor; TSS, transcriptional start site; UXT, ubiquitously expressed transcript.

To investigate the functional implications of the interaction observed between SENP1 and UXT, we carried out enzymatic and reporter assays. In the enzymatic assay, we monitored the ability of SENP1 to cleave off SUMO1 from AMC (SUMO1-7-Amido-4-methylcoumarine, SUMO1-AMC). For this, we used a catalytic active recombinant fragment of SENP1, SENP1[297–644], at 400 pM and different concentrations of recombinant UXT. The deSUMOylation activity was measured using a fluorometer plate reader resulting in end-point measurements of SENP1 activity (Fig. 7*A*) as well as using a fluorescence spectrophotometer to obtain a continuous measurement of SENP1 activity (Fig. 7*B*). Based on these enzymatic assays, the activity of SENP1 was gradually reduced in a UXT concentration-dependent manner. The activity of SENP1 remained unaffected when we used increasing amounts of GST (Fig. 7*A*), confirming that the effect is specific to UXT.

To supplement these *in vitro* assays with a comparable *in vivo* assay, we reasoned that since MYB is activated by SENP1 (24, 25), it should be possible to use this effect of SENP1 to monitor its inhibition by UXT *in vivo*. Therefore, we carried out MYB reporter assays in HEK293-C1 cells containing an array of Gal4-responsive elements (5×GRE), which is stably integrated in front of a luciferase reporter (70), and we used a MYB construct where its DBD is replaced by a Gal4p-DNA-binding domain (GBD). With this setup, SENP1 activated MYB several fold (Fig. 7*C*), allowing us to investigate the effect of transfecting an increasing amount of UXT on SENP1 activity by monitoring the activity of MYB on the luciferase reporter gene. Similar to the enzymatic assay, this also clearly showed that UXT attenuates the enzymatic activity of SENP1 (Fig. 7*C*). To control whether the changes in reporter activity is not due to changes in level of MYB, we measured the protein level of MYB in HEK293-C1 cells by WB analysis of the transfection setup used in Figure 7*C*. Although cotransfection with SENP1 increased the protein level of transfected GBD-MYB, no further effect on the levels of GBD-MYB expression was observed by increasing levels of UXT (Fig. S8*A*). When we investigated the effect of increasing amounts of UXT on a negative control, Gal-VP16, which was unaffected by SENP1, UXT had no similar inhibitory effect on the activity of VP16 (Fig. 7*D*).

To further show that the effect of UXT on MYB was dependent on SENP1, we extended the reporter assays in HAP1 cells reported in Figure 4, *C* and *D* by incorporating transfections with a plasmid encoding UXT-V5 in increasing concentrations. Again, transfection with increasing amounts of UXT led to gradual decrease of MYB’s activation function in the HAP1 cell line with WT levels of SENP1(Fig. 7*E*), whereas the activity of MYB was unaffected by UXT in the SENP1-KO cell line (Fig. 7*F*). This is consistent with our hypothesis that UXT requires the presence of SENP1 in order to inhibit the activity of MYB. This behavior also implies that the effects of UXT on MYB are caused by a change in MYB’s level of SUMOylation. To directly test this, the same cell lines were transfected with the 2KR version of MYB (SUMO conjugation–deficient MYB, see Experimental procedures and Fig. S1*A*). As expected, with MYB lacking SUMOylation sites, UXT had no inhibitory effect, neither in the HAP1 WT nor the SENP1-KO cell lines (Fig. 7, *G* and *H*, respectively). Taken together, we concluded that UXT attenuates the enzymatic activity of SENP1 both *in vitro* and *in vivo*. WB analysis after transfection setup in the HAP1 cells showed stable levels of MYB expression (Fig. S8*B*).

### UXT is also a target gene of MYB

Having found SENP1 to be a MYB target gene, we wondered whether its interaction partner also would be affected by MYB. Hence, we looked at UXT in the same dataset as for SENP1, and in fact, we found that UXT was upregulated upon endogenous MYB knockdown (Fig. 7*I*) and these phenotype was rescued by both the WT-, 2KR-, and ANAA-MYB rescues. By examining the ChIP-Seq occupancy of MYB at *UXT* locus, which showed a sharp and strong MYB ChIP-Seq peak at the TSS of *UXT* (Fig. 7*J*), we determined that similar to *SENP1*, *UXT* is also a direct target gene of MYB.

## Discussion

SUMOylation controls the activity of a large fraction of TFs and cofactors (1, 6, 9), implying SUMO-mediated global regulation of cellular processes. However, SUMOylation of clusters of TFs and other regulatory proteins combined with a balance between modification and demodification create an intricate interplay difficult to dissect. The TF MYB is an interesting model for SUMO control of gene expression, being subjected to both SUMO conjugation and SUMO binding (21, 22, 23, 24, 25). When we investigated how these SUMO-related features of MYB affected its downstream targets in K562 cells, we observed a large number of differentially regulated genes between WT MYB and MYB with an altered SUMO status (Figs. 1 and 2). In addition, we observed a distinct gene ontology term enrichment among the genes differentially regulated by the MYB mutants compared to the WT. This indicates that the SUMO status of MYB both quantitatively and qualitatively affect its regulome. Moreover, we observed that the two SUMO negative mutations of MYB, 2KR-MYB and ANAA-MYB, showed quite similar changes in expression patterns despite having distinct pattern of SUMO association (Fig. 1*D*). This probably relates to the concept of “group SUMOylation” where clusters of proteins undergo SUMOylation in a concerted manner and where SUMO–SIM interactions contribute as a glue to stabilize the clusters (reviewed in (16).

Recent studies have revealed that a key function of SUMO appears to stabilize cellular states, thus being important for cellular identity. Work from Dejean *et al.* has shown a drastic rewiring of the SUMOylome from embryonic stem cells to mouse embryonic fibroblasts and that SUMOylation acts as a general barrier to cell-fate changes (71, 72). Mechanistically, they concluded that SUMO functions as a tether or glue on distinct chromatin types to stabilize occupancy of key protein substrate complexes, thus preserving the somatic and pluripotent states (72). Studying the role of SUMO during adipocyte differentiation, Zhao *et al.* (73) similarly concluded that SUMO plays a key role in the transcriptional identity switch from pre-adipocyte to mature and functional adipocyte. Exactly how this function is exerted is not clear. The phenomenon of group SUMOylation–stabilizing assemblies probably plays a key role here, where networks of proteins assembled on chromatin are particularly important. In line with this, Palvimo’s lab recently showed for the glucocorticoid receptor (GR) that SUMOylation modulates the specificity of GR by regulating its chromatin protein network and accessibility at GR-bound enhancers (17). One aspect of this phenomenon may be seen also from our MYB data. Removing SUMO conjugation from MYB had diverse effects, as judged from which genes were affected, some clearly related to control of cell identity, which is largely controlled by transcription. A large fraction of the affected target genes were involved in transcriptional or epigenetic regulation (Fig. 1). In the set of novel target genes, seen in the 2KR-set, gene ontology-term analysis showed regulation of transcription and gene expression among the top enrichments (Fig. 2).

SUMO modification is often associated with transcriptional repression (4, 74, 75) or more precisely with limitation or modulation of gene activation (reviewed in (16)), but in some cases also, SUMO-mediated gene activation has been observed (76). One implication of this general pattern is that relieving the SUMO-mediated repression of transcription could in most cases contribute significantly to gene activation. In this way, SUMO proteases may play a role as putative activators of gene expression, as we have observed for MYB (24, 25). We therefore became interested in the role played by the key SUMO protease SENP1 in the MYB regulome. We focused on this protease because of its key role during hematopoiesis. SENP1^−/−^ embryos die at midgestation due to severe fetal anemia stemming from deficient erythropoietin production (50). SENP1 was also found to be essential for the development of early T and B cells (51).

Using transcriptome data as well as MYB ChIP-Seq data generated from K562 cells, we showed that SENP1 in fact is a MYB target gene (Fig. 4, *A* and *B*). SENP1 is also on the list of MYB target genes reported by Zhao *et al.* (77) using a truncated MYB for genome expression profiling. The consequence of this pattern would be that when the expression of MYB increases, it will act as a regulator of SENP1, leading to a shift in the balance of SUMOylation/deSUMOylation. Hence, its own level of SUMO modification would be expected to decrease. In this way, MYB may be able to modulate its own transcriptional activity as well as the activity of other factors affected by SUMOylation. In this model, by regulating the expression of SENP1, MYB indirectly modulates the SUMO landscape by shifting the balance between SUMOylation and deSUMOylation.

The intriguing implication is that MYB, by controlling the level of a key player in the SUMO system, may be subject to an autoactivation loop. In other words, as the expression level of MYB increases, its SUMO status should approach that of 2KR-MYB. A direct test of this hypothesis is challenging both since a gradual increase in MYB level is difficult to control and also because a quantitative measurement of SUMOylation levels is technically difficult. However, as an indirect assay, we tested the autoactivation loop hypothesis by performing a reporter assay in HAP1 SENP1-KO and the parental control cell lines. This showed that the endogenous level of SENP1 enhanced the activity of MYB (Fig. 4*C*) to a level comparable to that seen by the SUMOylation-deficient 2KR-MYB (Fig. 4*D*), which is constitutively active and whose activity is not dependent on SENP1-mediated deSUMOylation. In contrast, such enhancement of activation was not observed in the SENP1-KO cell line (Fig. 4*C*) supporting the autoactivation hypothesis. Moreover, the activation by deSUMOylation of MYB was unaffected in both cell lines when we used the SUMOylation-deficient 2KR-MYB (Fig. 4*D*).

Our findings may have implications for understanding oncogenic activation of the proto-oncogene MYB. In chicken, where c-*myb* was first discovered as the cellular homolog of the oncogene v-*myb*, oncogenic activation is caused by deletions in the negative regulatory domain of c-Myb. These deletions remove the SUMO-conjugation sites and make the v-myb protein more active. In humans, deletions in MYB are rare, but overexpression is common in several types of cancers. MYB gene amplification and overexpression have been observed in acute myeloid leukemia, non-Hodgkin lymphoma, colorectal cancer, and breast cancer (78, 79, 80, 81, 82, 83, 84, 85). One implication of the observations in the present report is that overexpressed MYB may be less restricted by SUMO conjugation than MYB expressed at lower levels due to its direct regulation of SENP1 expression. If this is the case, overexpressed MYB could be activated through reduced SUMOylation levels, not unlike what happens to v-myb because of its deletions.

There are an increasing number of reports about SUMOylation of proteins implicated in human cancer and diseases (62, 63, 64, 65, 66). In this regard, we found that overexpressed *MYB* correlates positively with *SENP1* expression in leukemia, breast, and colorectal cancer patients according to mining of data from TCGA (58) (Fig. 4, *E*–*G*). It is noteworthy that SENP1 was reported to be frequently overexpressed in human breast cancers, resulting in c-Myc stabilization and activation (67). Both *SENP1* and *MYC* are target genes activated by MYB (86, 87). Wang *et al.* (88) reported that SENP1 was overexpressed specifically in triple-negative breast cancer. Furthermore, they found that SENP1 is essential for triple-negative breast cancer cell proliferation and migration *in vitro*, as well as for tumor formation and metastasis *in vivo*. In addition to breast cancer, SENP1 overexpression has also been implicated in the development of bladder cancer, prostate cancer, neuroblastoma, osteosarcoma, and lung cancer (reviewed in (5)).

It may be argued that our conclusions on target genes affected by the SUMO status of MYB could be affected by hidden clonal variations (89, 90), since the transcriptome analyses are based on selected clones expressing mutants of MYB similar to physiological levels that are compared to the original cell population treated with control siRNA or MYB siRNA. One argument against this concern is the similarity of changes in gene expression patterns observed with 2KR-MYB and ANAA-MYB rescue, since it is highly unlikely that both these cellular clones would have been altered in the same manner. Moreover, such artefacts would not be reflected in cancer patient cohorts. However, when we examined three such cohorts for correlations between MYB expression and expression of target genes evaluated to be affected by SUMO status, a high percentage of positive correlations were found, consistent with the patterns found in K562 and with the autoactivation hypothesis.

Given this intriguing role of SENP1 in understanding MYB function, we also looked for SENP1-interaction partners. Using a stringent variant of Y2H screening, we identified UXT as a novel interaction partner of SENP1 (Fig. 5*A*) and verified the interaction by GST pull-down (Fig. 5*B*) and co-IP (Fig. 5*C*). We further investigated the biochemical and functional implication of the interaction between SENP1 and UXT using enzymatic assays (Fig. 7, *A* and *B*) and reporter assays (Fig. 7, *C* and *E*). These clearly showed that UXT acts as an inhibitor of SENP1 and that UXT’s indirect effect is on MYB activity and not MYB protein levels (Fig. S8).

UXT has been linked to several regulatory transcriptional processes such as transcriptional corepression of GATA4, FOG2, and other cardiac TFs leading to downregulation of cardiac specific genes (91). The protein act as a repressor of p53 through binding to MDMX, which suppresses the basal activity of p53 and leads to activation of the NF-kB (92). Moreover, UXT act as a repressor of Notch signaling (93) and as a coregulator of androgen receptor, regulating androgen-responsive genes (66). Another study coupled UXT to the SUMO pathway, where a physical interaction between UXT and PIAS2 was reported (94). The PIAS family acts as E3 ligases for SUMO conjugation (95). Whether the SENP1-repressive effect of UXT can explain some of these processes remains to be investigated. But UXT has also been linked to diverse processes beyond transcriptional regulation, such as mitochondrial aggregation, centrosome function, and apoptosis (60, 96, 97).

In this work, we further coupled UXT to transcriptional regulation, showing that UXT, like SENP1, is a target gene of MYB but in the opposite fashion being upregulated after MYB knockdown. Our MYB ChIP-Seq data supports direct regulation. Through this negative regulation of UXT by MYB and UXT acting as a SENP1 inhibitor, all the effects of SENP1 on MYB discussed above are expected to be strengthened by adding UXT to the regulatory circuit. The autoactivation loop will be kept up by two MYB targets, reinforcing each other in moving MYB in the MYB-2KR direction upon overexpression.

In conclusion, we showed that the SUMO status of MYB affects its regulome significantly both qualitatively and quantitatively. We identified MYB as transcriptional regulator of SENP1 and UXT, and UXT is identified as an inhibitor of SENP1, which defines MYB as a novel player in the SUMO system being able to modulate the SUMO landscape. Our findings further imply an autoactivation loop modulating MYBs’ own transcriptional activity. We propose that overexpressed MYB, as seen in multiple cancers, may drive this autoactivation loop and be a key element in the mechanism of oncogenic activation of MYB, having a similar effect as the classical oncogenic deletions in chicken c-Myb.

## Experimental procedures

### RNA-Seq and analysis

RNA-Seq analysis was performed after endogenous MYB knockdown on (i) K562 bulk cells with stable transfection of an empty vector (pEF1neo) (42) and (ii) single cell clones of K562 stably expressing either WT MYB (WT-MYB) or one of two MYB SUMO mutants, a SUMO conjugation negative mutant (2KR-MYB) and a SUMO-binding mutant (ANAA-MYB). These clones were selected to express levels of exogenous MYB close to endogenous levels in K562 (Fig. S1). Transfection of these K562 cells with siRNA (siU2992) and isolation of total RNA was made as we described in (42). The siRNA, siU2992 is designed to specifically target the 3′-UTR of the endogenous MYB mRNA. As a result, the exogenously introduced rescue MYB versions in each of these clones remain resistant to siRNA-mediated knockdown as they do not contain UTRs. Since the exogenous MYB is not knocked down, while the endogenous MYB is, we call this a rescue strategy. RNA samples were delivered for sequencing to the Norwegian sequencing center, where libraries were prepared using strand-specific TruSeq library prep kit. Transcriptome data for the cell lines with three biological replicates were generated using Illumina HiSeq 4000 sequencer, where 150 bp paired-end reads were obtained. The present transcriptome data along with our previously generated transcriptome data (GSE85187) from (42) was analyzed. Quality control, differential expression, and downstream bioinformatics analysis was made as described in (15). Transcriptome-wide fold changes of the differentially expressed genes between the various groups (KD, ctrl, WT-MYB-rescue, and various MYB mutant rescues) are presented in volcano plots (Fig. S2).

GSEA of biological processes as well as pathway and diseases enrichment analysis against the KEGG database was made using MSigDB database v6.1 (98). In the GSEA, only terms that have Benjamini and Hochberg adjusted *p* value <0.05 were included.

### K562 ChIP-Seq data

We utilized our recent ChIP-Seq data from (45). We expanded our list of MYB occupied genes described by Lemma and Ledsaak *et al.* (45), where in addition to the original association of MYB ChIP-Seq peaks with regulatory element to gene links made with the STITCHIT algorithm (99), we introduced a distance-based approach to assign peaks to their nearest TSS as described in (100). Here, we utilized the HOMER annotatePeaks.pl script with default setting (101).

### MYB correlations with SENP1 and other 2KR-MYB target genes in TCGA data

We utilized RNA-Seq data from leukemia, breast, and colorectal cancer patients, LAML-US, BRCA-US, and COAD-US TCGA cohorts, respectively (58). We computed Pearson correlations between MYB and SENP1 expressions in the respective cohorts using the *stat_cor* R function with the parameter *method = “pearson*” using the *ggscatter* function in *ggplot2*. The systematic correlation analysis between MYB expression and every 2KR-MYB target genes in LAML-US, BRCA-US, and COAD-US cohorts was performed with the *cor.test* R function using an automated snakemake pipeline.

### Y2H screening

The Y2H screen was done on a human thymus cDNA library using a centromeric bait plasmid for obtaining single-copy (CEN-plasmid approach) as described in (15, 39, 102, 103, 104).

### Plasmid construction

A cDNA encoding UXT variant 2 was cloned into various expression vectors using the Gateway cloning system. First, *UXT* was PCR-cloned into the donor vector pDONR122 (Invitrogen) *via* the BP reaction using oligos UXTgwFrw: 5′-GGG GACAAGTTTGTACAAAAAAGCAGGCTTCATGGCGACGCCCCCTAAGCG-3′ and UXTgwRev: 5′-GGGGACCACTTTGTACAAGAAAGCTGGGTTCAATGGTGAGGCTTCTCTG-3′, followed by transferring *UXT* into two destination vectors, pGEX-AB-GAW (for recombinant protein expression) and pDEST-HA (for mammalian expression) *via* the LR reaction. A C-terminal V5 tag (DNA technology) was cloned into pCIneo (Promega) using XhoI and NotI. The oligos for V5 tag, V5-oligo-U: 5′-TCGAGATATCCGCGGATCCGGCAAGCCTATCCCTAACCCTCTCCTCGGTCTCGATTCTACGTAGC-3′ and V5-oligo-L: 5′-GCCGCTACGTAGAATCGAGACCGAGGAGAGGGTTAGGGATAGGCTTGCCGGATCCGCGGATATC-3′ were ligated by annealing equal volumes of the two oligos as follows (95 °C for 5 min and 15 min at room temperature). *UXT* was PCR-cloned with oligos UXT-F-XhoI: 5′-actacttcgctcgagccaccATGGCGACGCCCCCTAAGCGG-3′ and UXT-R-SacII: 5′-acatgatccgcgggtaccATGGTGAGGCTTCTCTGGGA-3′ into pCIneo-V5 using XhoI and SacII resulting in a C terminally V5-tagged UXT (pCIneo-UXT-V5) construct. We used AlwNI and NheI to transfer the UXT-V5 fragment into another mammalian expression vector, pEF1neo, where the human cytomegalovirus promoter from pCIneo is replaced with the human EF1-alpha promoter. The resulting mammalian expression construct pEF1neo-UXT-V5 was used for stable K562 cell line generation. Recombinant GST-UXT-V5 fusion protein was made by transferring *UXT-V5* from pCIneo-UXT-V5 to pGEX-6P2 (GE Healthcare) using XhoI and EagI. The mammalian expression vectors pCIneo-3×FLAG-hcM and pCIneo-3×FLAG-hcM-2KR are described in (25). Constructs that were used in reporter assays, pCIneo-GBD2-VP16 that encodes the Gal4 DBD in fusion with the herpes simplex virus VP16 transactivation domain is described in (105), pCIneoB-GBD2-hcM [194–640] is described in (106), MYB-responsive reporter plasmid pGL4b-3×MRE(GG)-MYC-aab, which contains human MYC P2 core promoter sequence and three MREs, is described in (24).

### Cell culture, stable cell line generation, and luciferase assay

In this work, we used four cell lines: K562 (ATCC CCL-243 *Homo sapiens* bone marrow, chronic myelogenous leukemia), HEK293-C1 (ATCC CRL-1573 *H. sapiens* embryonic kidney), COS-1 (ATCC CRL-1650 Cercopithecus aethiops kidney), and HAP1 cell lines (Horizon). The HAP1 SENP1 KO cell line, the HAP1 control cell line, and the K562 cell lines were maintained as described in (15). HEK293-C1 and COS-1 cells were maintained as described in (24). All cells were grown at 37 °C and 5% CO_2_. Transient transfections of COS-1, HEK-293-C1, and HAP1 cells with the indicated plasmids were made using the TransIT-LT transfection reagent (Mirus Bio) as described in (39). K562 single cell clones stably expressing a C-terminal V5-tagged UXT (pEF1neo-UXT-V5) or an empty vector (pEF1neo-V5), N-terminally 3×FLAG-tagged, and C-terminally HA-tagged MYB variants (3×FLAG-2KR-MYB-HA or 3×FLAG-ANAA-MYB-HA) were generated through transfection of the cells with the indicated plasmids by electroporation, and selection for single clones was performed as described in (45). Western blotting was used to verify positive clones that show stable expression (Fig. S1*B*). For the RNA-Seq data generation, we used 2KR-MYB clone 18 and ANAA-MYB clone 5. Reporter assays were carried out in transiently transfected HEK-293-C1 cells with a stable integration of 5×Gal4-luciferase reporter and HAP1 cell lines. The reporter assays were carried out in triplicates on 24-well trays that were seeded with 3.2 × 10^4^ HEK293-C1 or 5 × 10^4^ HAP1 cells per well using Luciferase Assay Reagent (Promega). The experiment was repeated at least in three independent experiments.

### Immunofluorescence

Immunofluorescence was carried out by seeding 1 × 10^6^ K562 cells that stably express UXT with a C-terminal V5 tag on glass slides in quadri-perm dishes. The glass slides were coated with poly-L-lysine solution (SIGMA) for 20 min prior to seeding. The cells were fixed with 4% paraformaldehyde solution for 30 min at room temperature 24 h after, and the slides were incubated with PBS containing 0.25% Triton X-100 for 4 min. Nonspecific binding of antibodies were blocked by incubating the cells with 1% bovine serum albumin (BSA) in PBS for 30 min followed by incubations with primary and secondary antibodies. Counterstaining was made with (4′,6-diamidino-2-phenylindole in a vectashield mounting medium (Vector Laboratories), and the cells were examined under an inverted confocal microscope (Olympus Fluoview 1000). The microscopy images were acquisitioned with the Image J2 (FIJI) software (https://fiji.sc/).

### Expression and purification of GST-fusion proteins

Expression and purification of GST-fusion proteins is detailed in Supporting information.

### GST pull-down and co-IP

GST fusion proteins and GST were expressed and isolated as described in (15). GST pull-down assay was performed as described in (15) except that the KAc interaction buffer was supplemented with 5× complete protease inhibitor cocktail (Roche Applied Science).

For co-IP, COS-1 cells in a 15 cm dish transfected with the indicated plasmids were harvested 24 h after transfection. Whole cell lysates were prepared, and the co-IP was performed as described in (15).

Co-IP at an endogenous level of SENP1 with UXT-V5 was made by incubating mouse anti-V5 monoclonal antibody (Invitrogen) coupled protein G Dynabeads (Invitrogen) with nuclear extract derived from K562 cells having stable expression of UXT-V5. Nuclear extract from the empty vector containing only the V5 tag in the K562 stable cell line was used as a control. Further detail about the endogenous IP is described in (15).

### K562 nuclear extract preparation and Superose-6 fractionation of the nuclear extracts

Nuclear extracts from K562 cell lines stably expressing the C terminally V5-tagged UXT were prepared as described in (107) with a slight modification detailed in (100). The nuclear extracts were desalted using HiTrap desalting column (Pharmacia Biotech) and fractionated using superose-6 columns as described in (15).

### Antibodies

For WB detection, we used the following primary antibodies: mouse anti-V5 monoclonal antibody (46-0705, Invitrogen), mouse anti-FLAG M2 monoclonal antibody (F3165, Sigma-Aldrich), rabbit anti-HA polyclonal antibody (H6908, Sigma), rabbit anti-SENP1 polyclonal antibody (ab108981, Abcam), mouse anti-GAPDH monoclonal antibody (AM4300, Invitrogen), goat anti-GAPDH antibody (NB-300-320, Novus biologicals), mouse anti-MYB (5E11) antibody (ab10934, Abcam), and rabbit anti-MYB H141 (sc7874, Santa Cruz Biotechnology). The following secondary antibodies were used for WB: anti-rabbit IgG-HRP (711-03-152, Jackson Immuno-Research), anti-mouse, anti-goat and anti-rabbit IRDye 680 RD (926-68072, 926-68074, and 926-68073, respectively), and anti-mouse IRDye 800 CW (926-32212) (LI-COR).

For immunoprecipitation, we used the following antibodies: FLAG M2 magnetic beads (Sigma), protein G Dynabeads (10004D, Invitrogen), rabbit anti-HA (H6908, Sigma), and mouse anti-V5 monoclonal antibody (46-0705, Invitrogen).

For immunofluorescence, we used the following antibodies: mouse anti-V5 monoclonal antibody (46-0705, Invitrogen), rabbit anti-SENP1 polyclonal antibody (ab108981, Abcam), Alexa Flour 488 anti-rabbit IgG (Invitrogen), and Alexa Flour 647 anti-mouse IgG (Invitrogen).

### Interaction site mapping using peptide arrays

Peptide array of SENP1 on a nitrocellulose membrane was made at the department of Biochemistry, University of Oslo. The membrane was blocked with 2% BSA for 1 h. This was followed by overnight incubation at 4 °C with 10 ng purified GST-fused recombinant UXT protein with C-terminal V5 tag (GST-UXT-V5) in lysis buffer (20 mM Hepes pH 7.6, 10% glycerol, 0.2% Triton X-100, 150 mM KAc, 1 mM DTT supplemented with 5× Complete protease inhibitor cocktail (Roche Applied Science)) supplemented with 2 mM DTT. The membrane was subjected to three washing steps with lysis buffer containing 1% BSA (each wash for 10 min) and was further blocked for 1 h at room temperature with TBS Odyssey blocking buffer (LI-COR). The array was probed with primary and secondary antibody followed by scanning with Odyssey CLx scanner (LI-COR).

### *In vitro* deSUMOylation assay

*In vitro* deSUMOylation assay was carried out in a 96-well plate in a 10 μl reaction mixture containing 5 μM SUMO1-AMC (UL-551, Boston Biochem), GST-SENP1 (400 pM), GST-UXT (various concentrations), and assay buffer (50 mM Tris–HCl pH 7.8, 100 μg/μl BSA, and 10 mM DTT). GST protein in varying concentrations was used as a negative control. The reaction was measured in a fluorometer plate reader Wallac Victor2 (PerkinElmer). The concentration of the fusion proteins was adjusted using SENP buffer (30 mM Tris–HCl, 100 mM KCl, 5 mM MgCl2, and 2 mM DTT). In addition to end-point enzymatic activity measurement, a continuous measurement of SENP1 enzymatic activity was made using LS50B Luminescence spectrometer (PerkinElmer) in a reaction mixture containing SENP1 at a concentration of 1000 pM, SUMO1-AMC at a concentration of 5 μM and varying concentrations of UXT.

### Data and code availability

The newly generated RNA-Seq data both raw and processed are available at GEO with the accession number GSE124542. The code used to process the RNA-Seq data is provided at https://github.com/rblemma/MYB_SUMO_status_transcriptome. The code used to generate the TCGA Pearson correlation tables (Tables S3 and S4) is provided at https://github.com/rblemma/MYB.vs.2KR-MYB_target_corrs_in_TCGA.

## Supporting information

This article contains supporting information (45, 68, 69, 108, 109, 110, 111, 112, 113, 114, 115, 116, 117, 118, 119, 120, 121, 122).

## Conflict of interest

The authors declare that they have no conflicts of interest with the contents of this article.
