# Supplementary material for: MYB regulates the SUMO protease SENP1 and its novel interaction partner UXT, modulating MYB target genes and the SUMO landscape
Source: J Biol Chem. 2023 Jul 17;299(9):105062. doi: 10.1016/j.jbc.2023.105062 (PMC10463205; doi:10.1016/j.jbc.2023.105062)
Supplement: Supporting Information [file mmc1.pdf]

Supporting Information

MYB regulates the SUMO protease SENP1 and its novel interaction partner UXT, modulating MYB target genes and the SUMO landscape

Running title: MYB inversely regulates SENP1 and its inhibitor UXT

Roza Berhanu Lemma[1,2*], Marit Ledsaak[1,3], Bettina Maria Fuglerud[1], Fernando Rodríguez-Castañeda[1], Ragnhild Eskeland[1,3,4], Odd Stokke Gabrielsen[1]

[1] Department of Biosciences, University of Oslo, P.O.Box 1066 Blindern, N-0316 Oslo, Norway

[2] Centre for Molecular Medicine Norway (NCMM), Nordic EMBL Partnership, University of Oslo, 0318 Oslo, Norway.

[3] Department of Molecular Medicine, Institute of Basic Medical Sciences, Faculty of Medicine, University of Oslo, PO Box 1112 Blindern, 0317 Oslo, Norway.

[4] Centre for Cancer Cell Reprogramming, Institute of Clinical Medicine, Faculty of Medicine, University of Oslo, Oslo, Norway.

[*]Corresponding author: Email: r.b.lemma@ncmm.uio.no

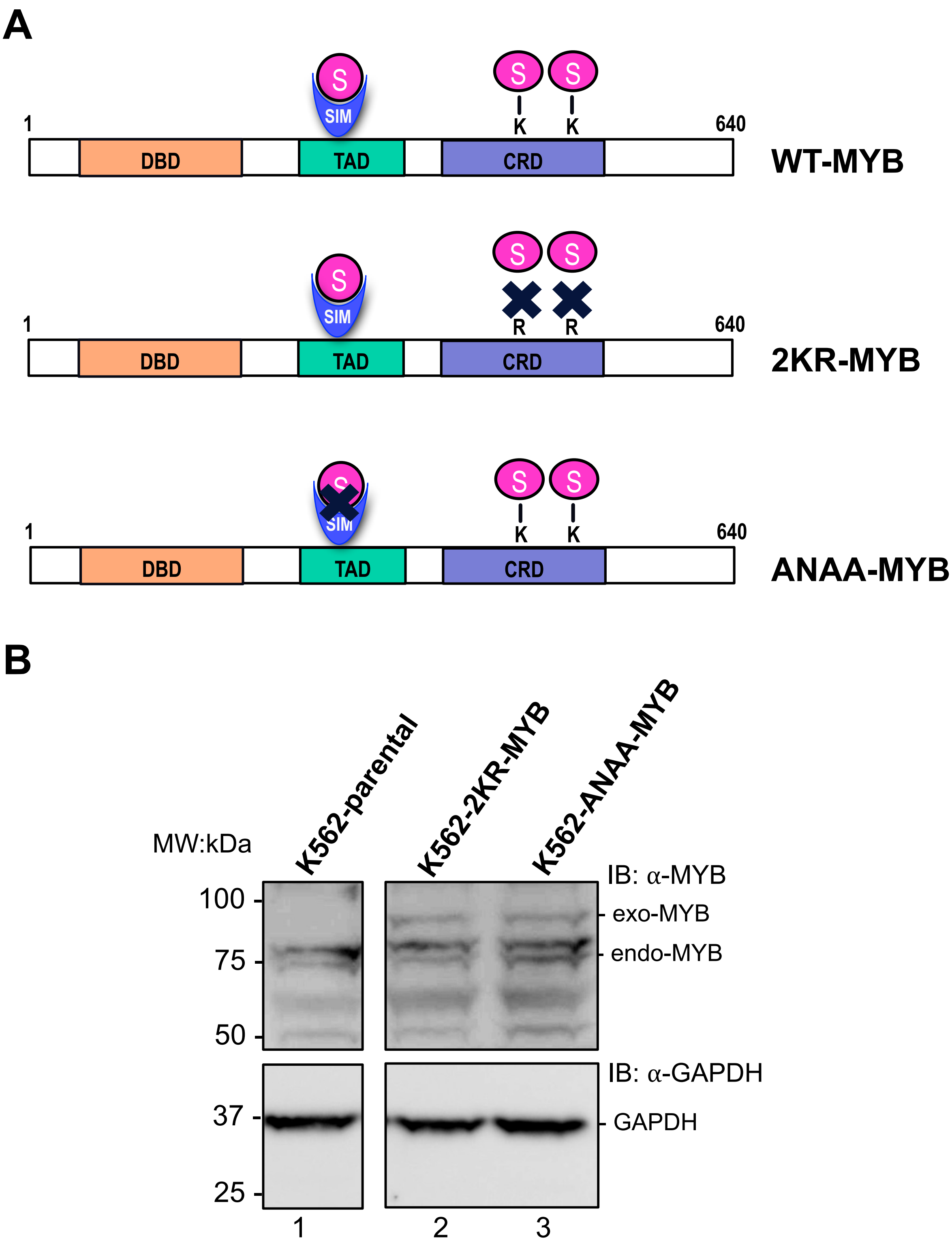

Figure S1. (A) Overview of the relative locations of the SUMO mutations introduced on the MYB sequence. The **top panel (WT-MYB)** shows the full length Wild Type (WT) MYB along with the two SUMO conjugation sites (K503 and K527) within the C-terminal regulatory domain (CRD) of MYB. SUMO proteins (purple circles) conjugated to the lysine residues (K503 and

K527) are displayed. In addition, we displayed the location of the SUMO Interaction Motif (SIM) represented with an inverted blue arch. The SIM is composed of residues, V_{267}NIV within the Trans-activation domain (TAD) of MYB with which a SUMO protein (purple circle) is bound to. The **middle panel (2KR-MYB)** shows the positions of the SUMO conjugation negative mutations. The SUMO conjugation sites (K503 and K527) are mutated into Arginine (R) abrogating SUMO conjugation. The **bottom panel (ANAA-MYB)**, shows the SUMO-binding site within the SIM, where it is mutated into A_{267}NAA abrogating SUMO binding. **(B) Characterization of MYB SUMO-deficient mutant used for generating the RNA-Seq data.** The figure shows western blot analysis of 2KR-MYB and ANAA-MYB expressions derived from cell lines from single clones of the corresponding stably integrated exogenous MYB in comparison with the endogenous MYB levels. Anti-MYB H141 (rabbit) antibody was used to detect the MYB levels.

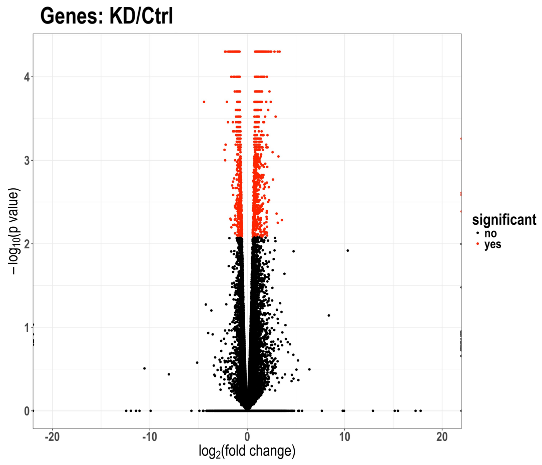
Genes: KD/Ctrl
−log10(p value)
log2(fold change)
significant
no
yes

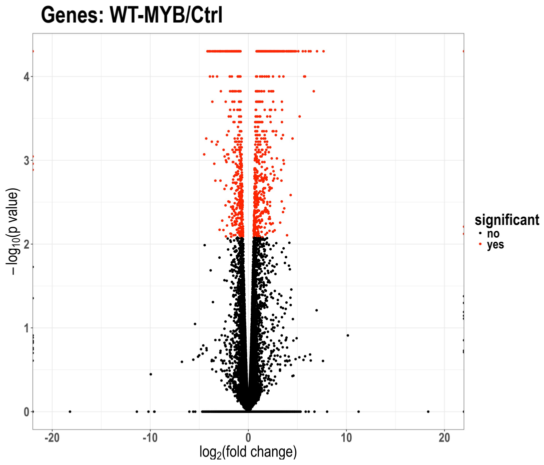
Genes: WT-MYB/Ctrl
−log10(p value)
log2(fold change)
significant
no
yes

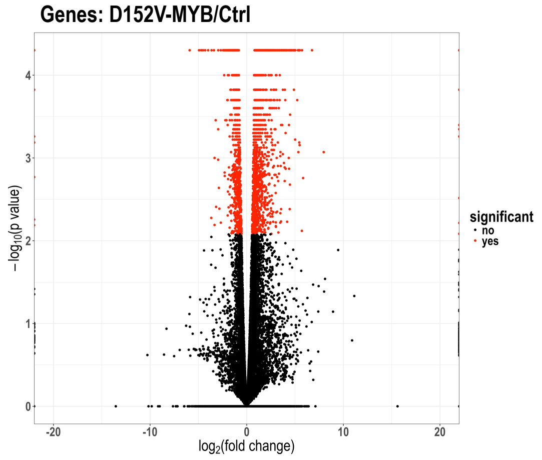
Genes: D152V-MYB/Ctrl
−log10(p value)
log2(fold change)
significant
no
yes

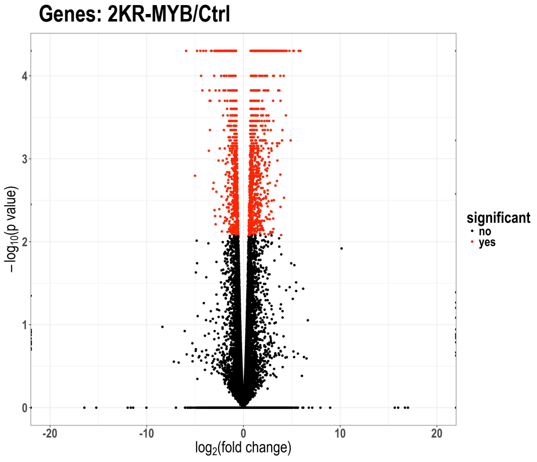
Genes: 2KR-MYB/Ctrl
−log10(p value)
log2(fold change)
significant
no
yes

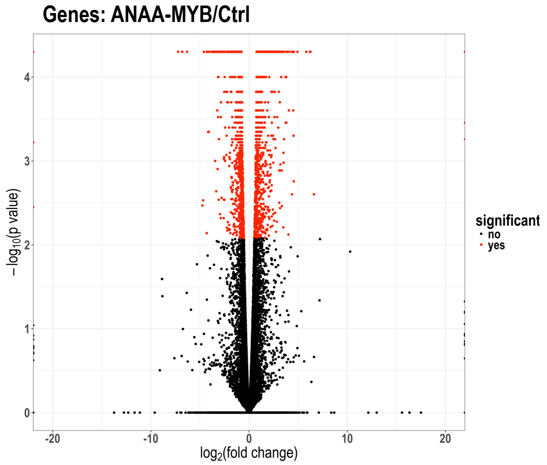
Genes: ANAA-MYB/Ctrl
−log10(p value)
log2(fold change)
significant
no
yes

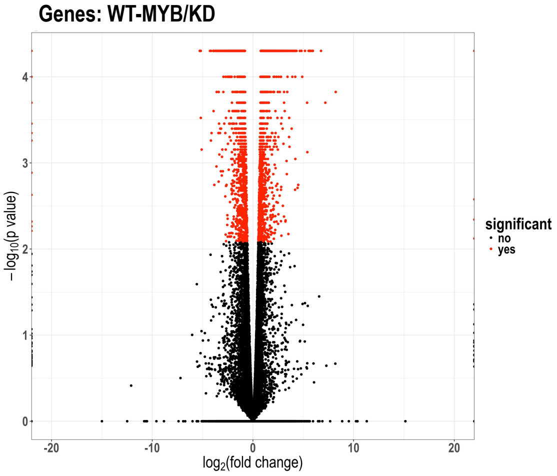
Genes: WT-MYB/KD
−log10(p value)
log2(fold change)
significant
no
yes

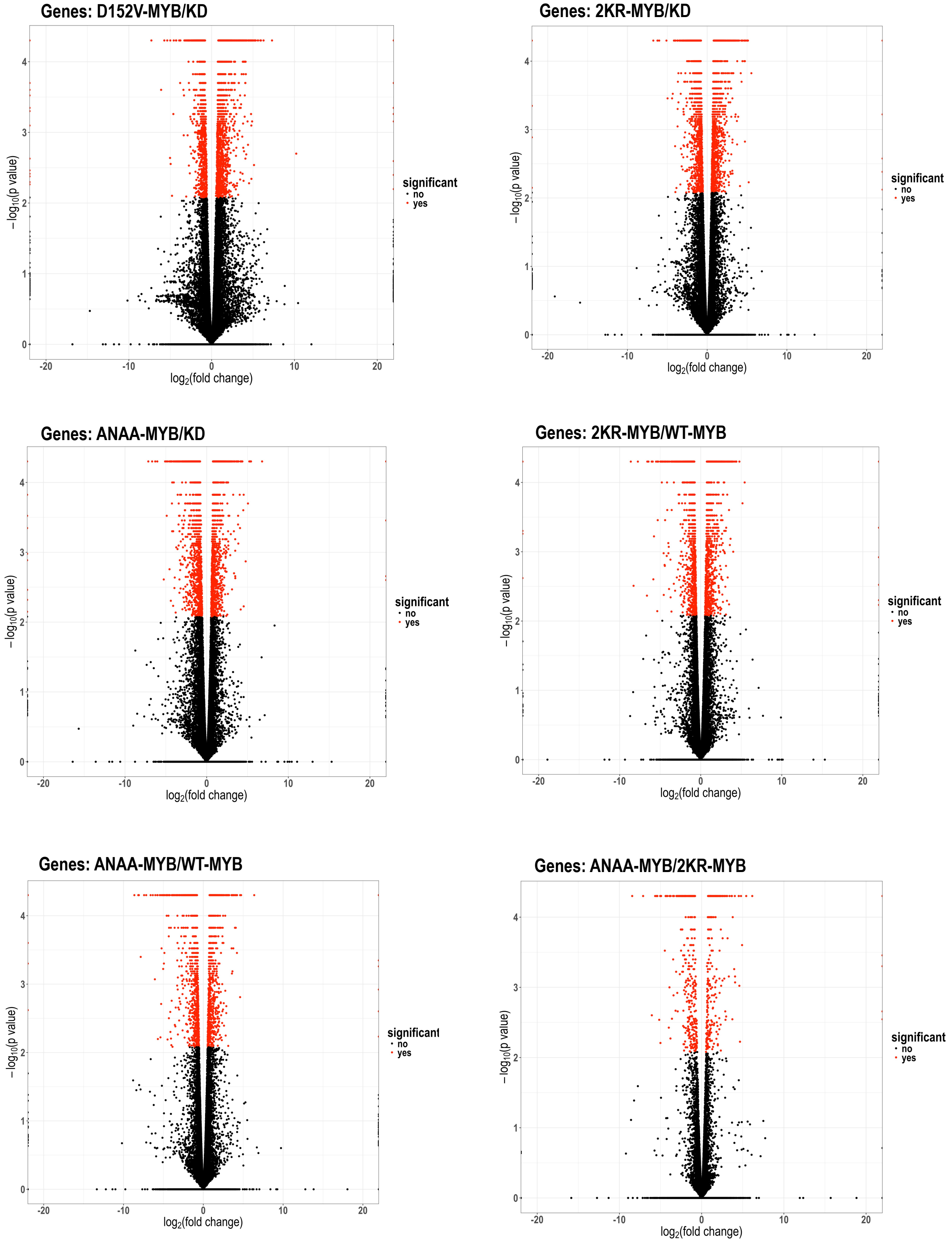

Figure S2. Differentially expressed genes for the endogenous MYB-KD and rescue of this KD with the different SUMO negative and SUMO positive versions of MYB. The different MYB

rescues were visualized in volcano plots relative to the transcriptome of K562 cells treated with a Ctrl siRNA, relative to the transcriptome of the endogenous MYB KD and relative to each other. Each gene is represented by one point. Fold change is indicated on the x-axis, whereas statistical significance is shown on the y-axis. Those genes that pass p-value < 0.01 significance threshold (represented by red points) were reported by cuffdiff as significantly differentially expressed (sigDE-genes). We have further filtered this sigDE-genes based on their log2FC q-value to control for FDR (false discovery rate), those genes with log2FC q-value < 0.01 are included for further analyses. Volcano plots were generated using the R package CummeRbund (Goff, L., C. Trapnell, and D.R. Kelley, *cummeRbund: Analysis, exploration, manipulation, and visualization of Cufflinks high-throughput sequencing data*. R package version 2.16.0. 2013).

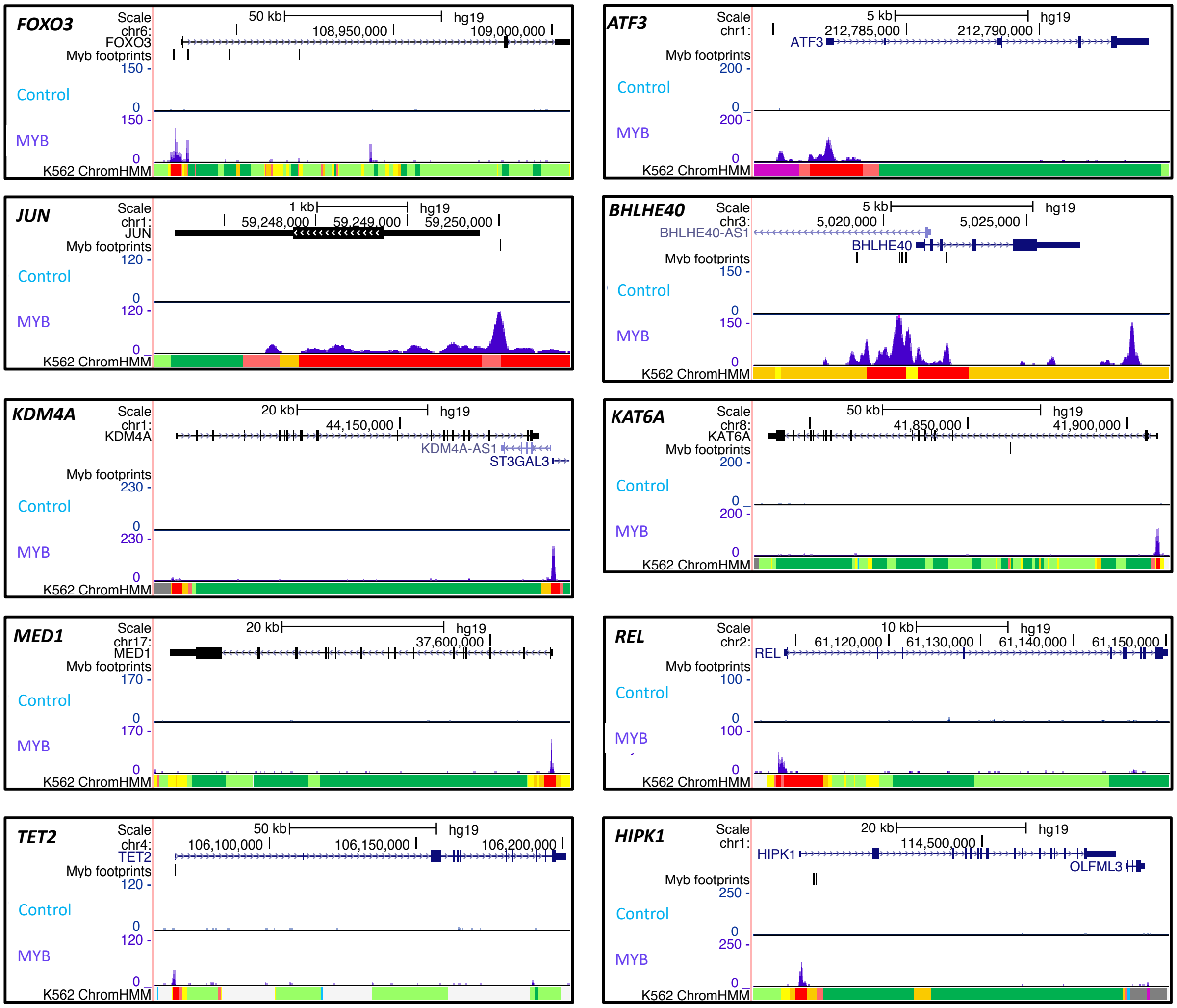

Figure S3. MYB occupancy the loci of selected target genes affected by SUMO status of MYB. MYB K562 ChIP-seq data from Lemma et al., 2021 [1] and K562 chromHMM chromatin segmentation tracks were visualize at the loci of selected MYB target genes that were significantly differentially regulated by the SUMO negative versions of MYB compared to wild-type MYB. Visualization of the tracks were made using the UCSC genome browser (https://genome.ucsc.edu/) [2] by creating a UCSC session containing the K562 control and MYB ChIP-seq bigwig files from Lemma et al. along with chromHMM segmentation track from K562.

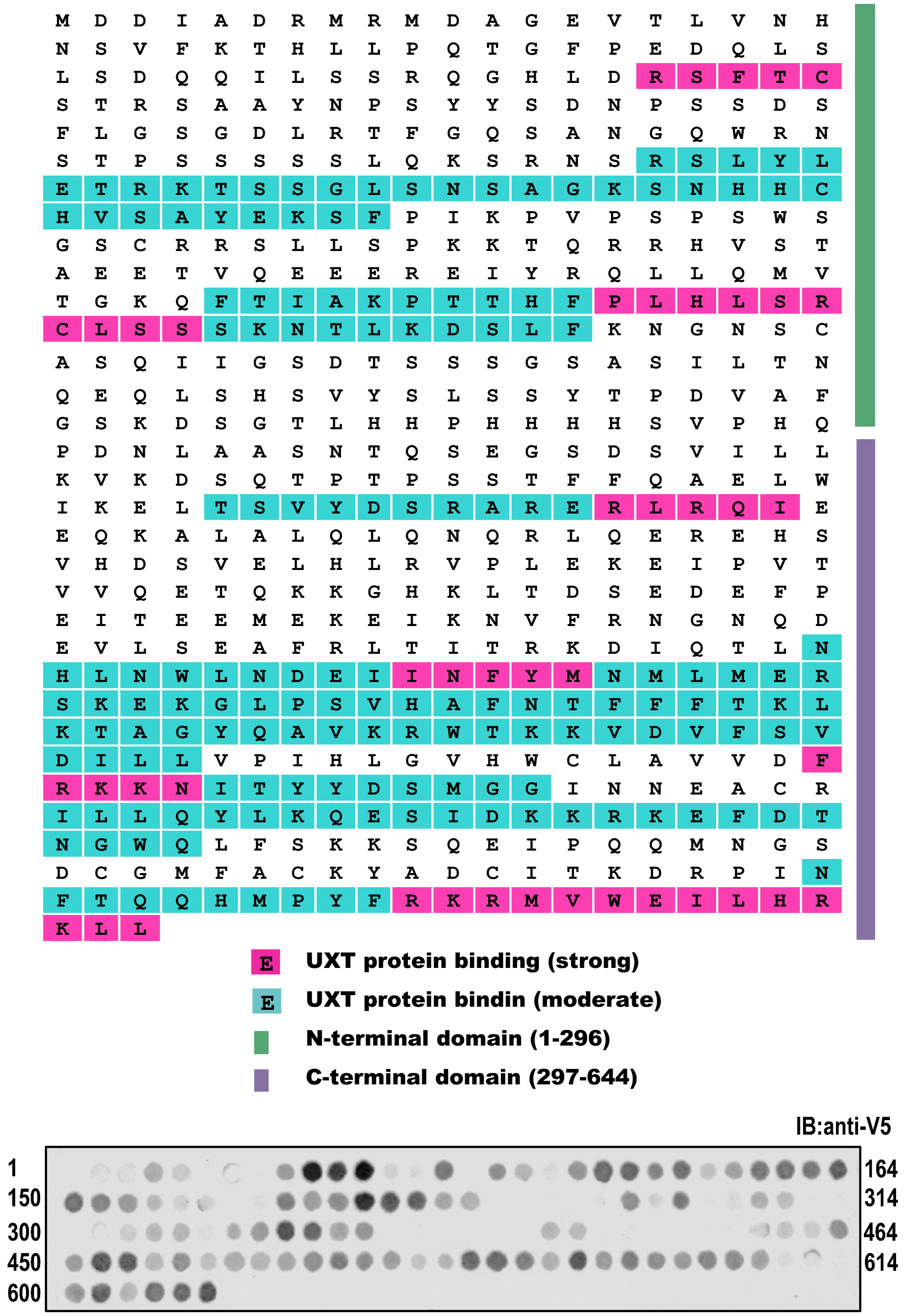

Figure S4. Interaction mapping using SENP1 peptide array. Upper panel shows the sequence information of the SENP1 peptide array along with UXT binding information. Lower panel shows the peptide array map of UXT-V5.

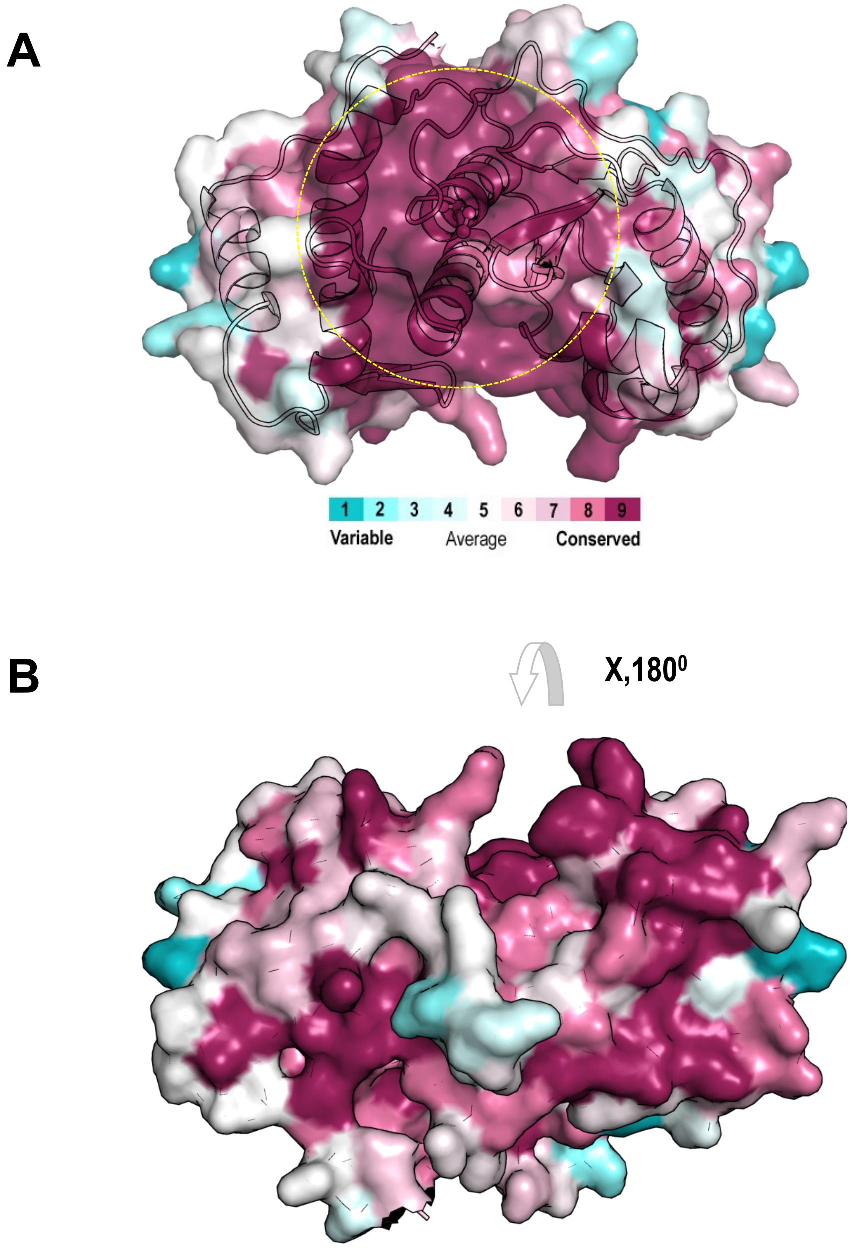

Figure S5. Evolutionary sequence conservation map. (A) A patch of highly evolutionarily conserved surface exposed residues of SENP1. The yellow circle around the patch shows a region in the sequence composed of residues 441-644. Homolog sequences of the human SENP1 protein sequences in metazoans was obtained from NCBI's Nr. Database using DELTA-BLAST and Expresso (www.tcofee.org) was used to generate MSA using the SENP1 experimentally solved structure (PDB ID: 2IY1) as a template. The resulting SENP1 MSA was used to calculate evolutionary amino acid sequence conservation by employing the Consurf Server [3-7]. The evolutionary amino acid sequence conservation was mapped on top of the experimentally solved SENP1 structure (PDB ID: 2IY1). Example of the Expresso alignment for fewer selected human SENP1 homolog sequences is shown in Figure S7. **(B)** The same figure as "(A)" where the structure was rotated 180° along the x-axis.

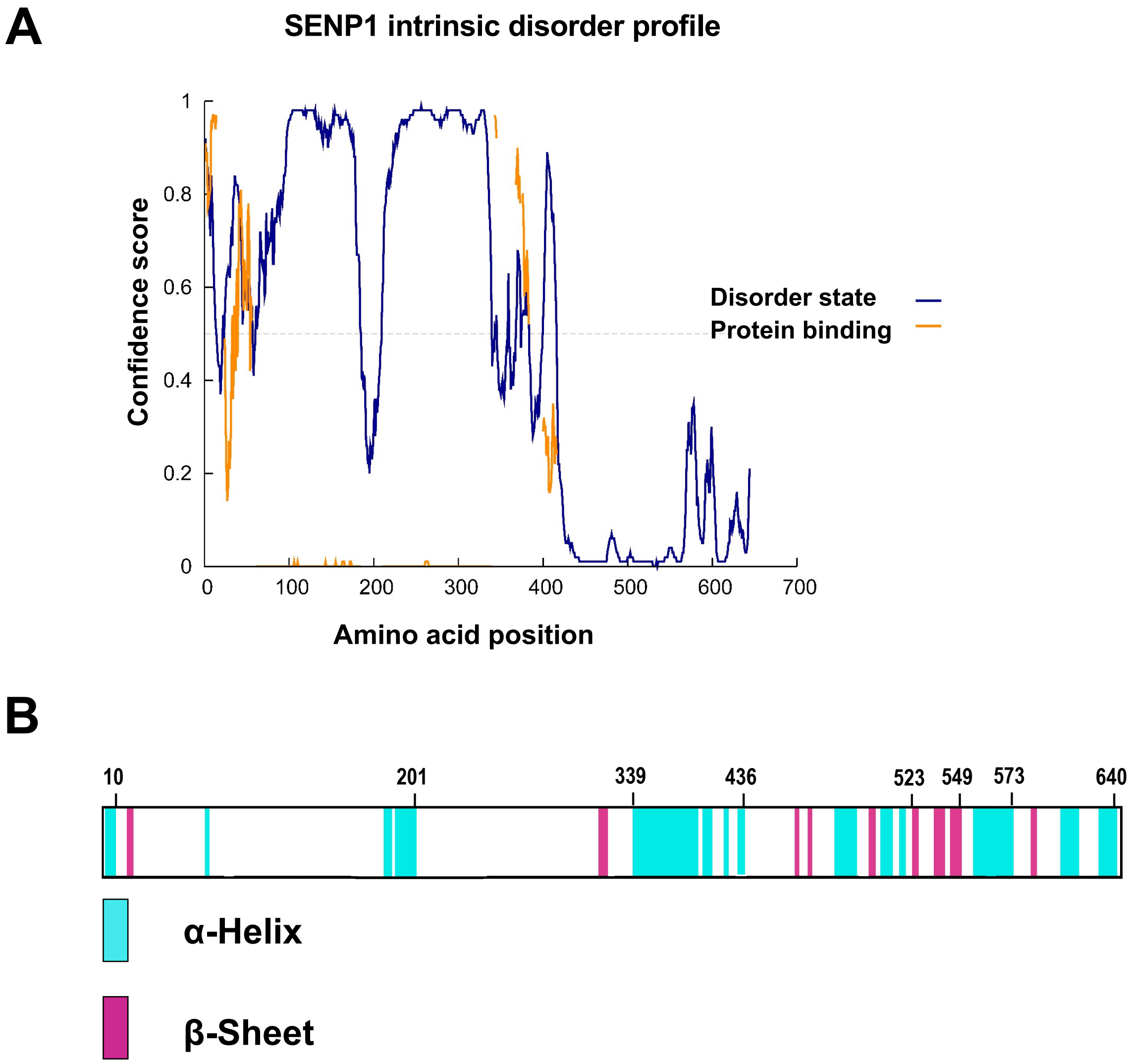

Figure S6. SENP1 disorder and secondary structure prediction. (A) Human SENP1 intrinsic disorder profile. DISOPRED3 [8, 9] was employed to predict intrinsic disorder for SENP1 protein structure. The profile shows the amino acid position in the x-axis and confidence score in the y-axis. Everything above the grey dotted line is predicted to be disordered. In the disorder state prediction, the orange profile is predicted to be protein binding. **(B)** Secondary structure prediction for the human SENP1 protein. Secondary structure prediction was performed using PSIPRED (v.3.3) [9, 10].The secondary structures and AA positions are displayed to scale.

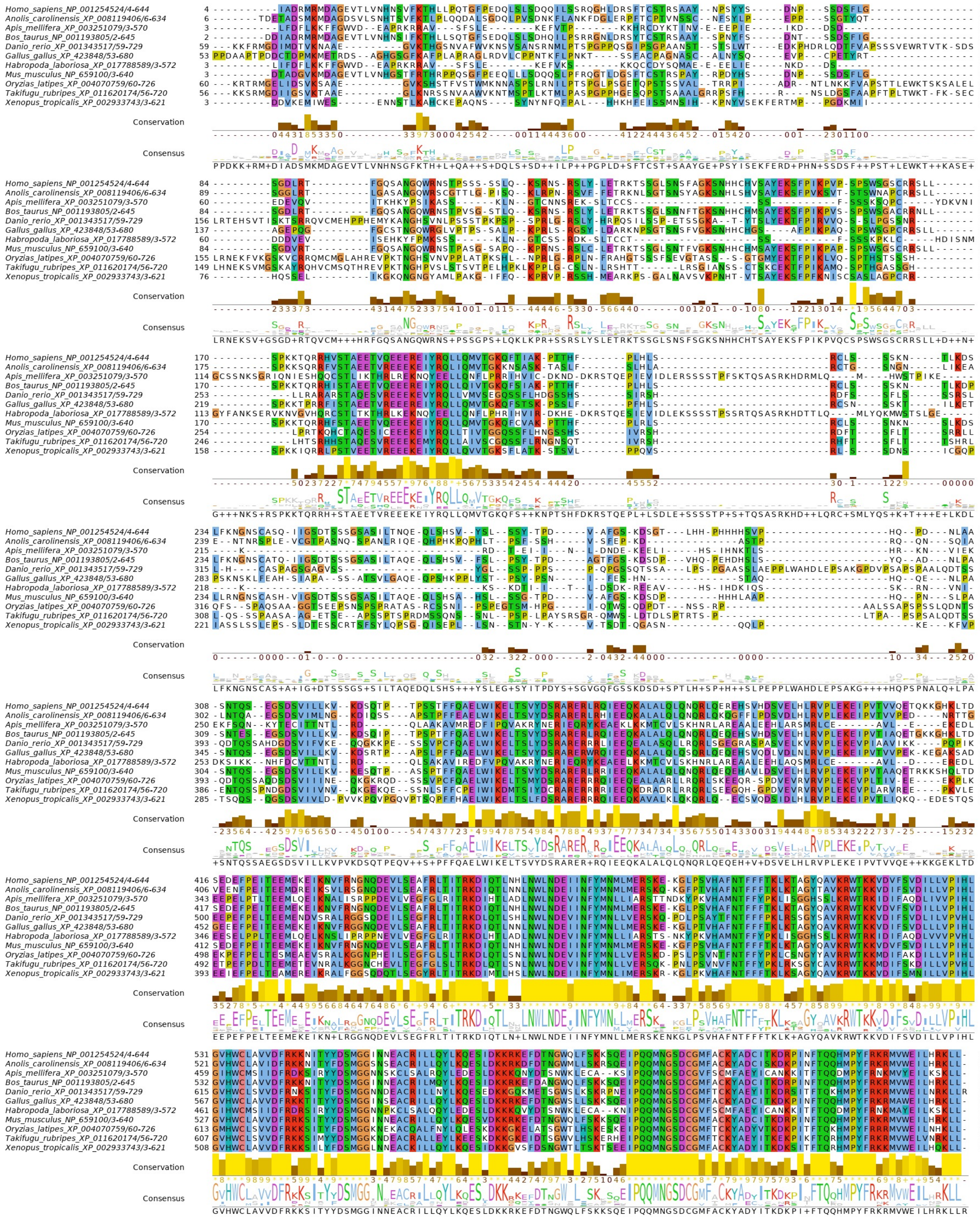

Figure S7. MSA of the human SENP1 homolog sequenced generated using the Expresso alignment. Expresso (www.tcofee.org) was used to generate MSA by using the SENP1 structure (PDB ID: 2IY1) as a template. Due to the large size of the generated MSA, only a few representative homolog sequences of SENP1 are shown in this figure. Visualization of the MSA was made using Jalview (v2.10) [11].

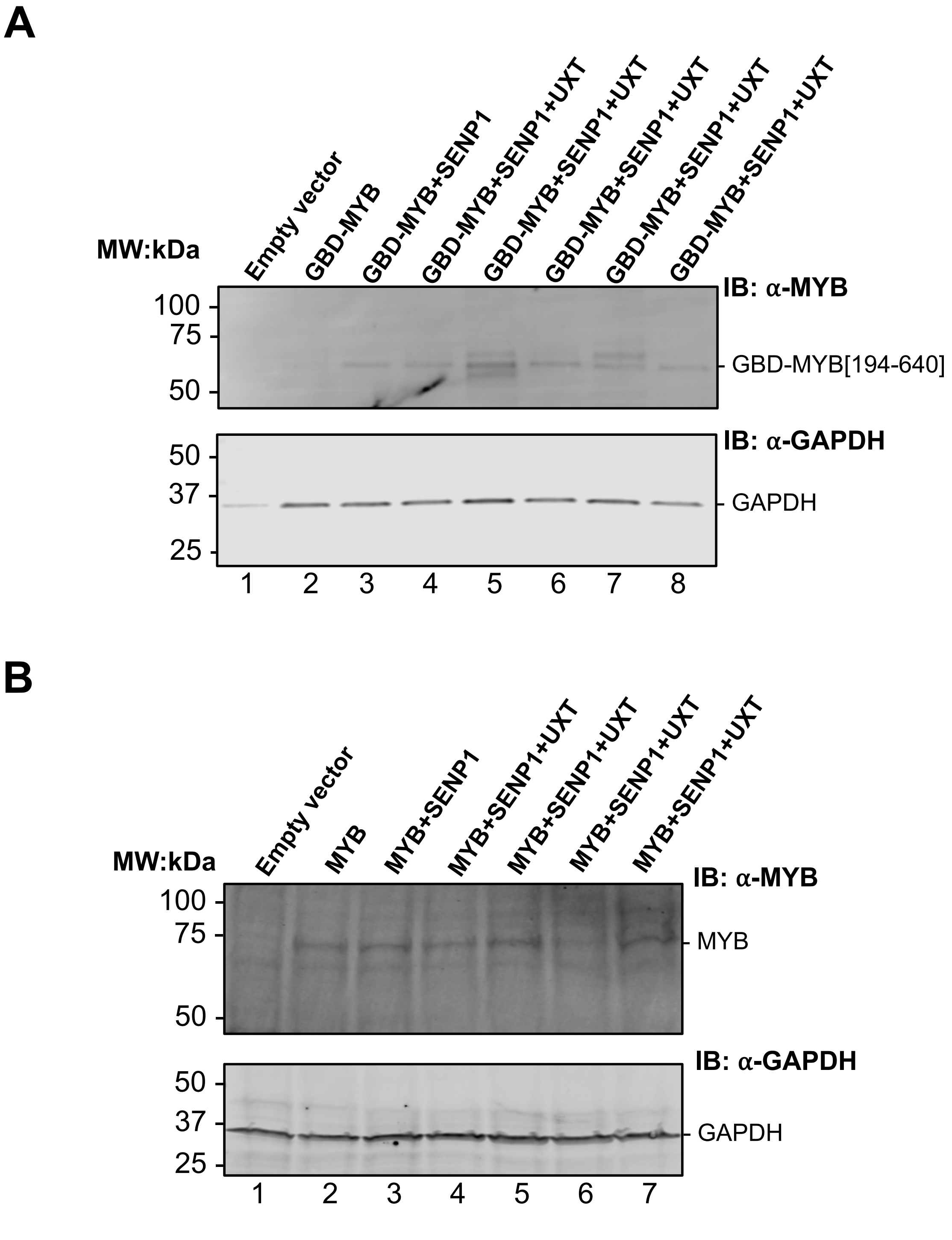

Figure S8. Western blot analysis of MYB transfection levels used for the reporter assays in Fig. 7. **(A)** Western blot analysis of GBD-MYB transfection levels corresponding to the reporter assay in Fig. 7C. We used mouse anti-MYB (5E11) antibody to detect MYB and goat anti-GAPDH antibody as loading control. **(B)** Western blot analysis of 3xFLAG-MYB transfection levels corresponding to the reporter assay in Fig. 7E. We used mouse anti-FLAG antibody to detect MYB and goat anti-GAPDH antibody as loading control.

Supplementary Results

Solvent exposed residues of SENP1 are evolutionarily conserved

In order to investigate the possibility of UXT binding to linear epitope in the disordered N-terminal domain of SENP1 and a 3D-epitope in the structured part (C-terminal domain), we used a combination of bioinformatics predictions together with our pull down and peptide array results. The bioinformatics approaches include sequence comparison, homology modelling, secondary structure and disorder prediction approaches along with AlphaFold's full length SENP1 structure prediction.

Using these approaches, we first investigated the amino acid conservation of SENP1 residues in a structural context. Specific amino acid residues are considered potential interaction sites, if they are found to be solvent exposed and highly evolutionarily conserved [12]. Therefore, we investigated the amino acid conservation of SENP1 and UXT across several metazoan species using sequence searches and multiple sequence alignments (MSAs). We then performed loop modelling of an already experimentally solved SENP1 structure (PDB ID: 2IY1), to have a full overview of the structure of this protein and mapped evolutionary sequence conservation on the modelled structure. This showed that most of the solvent exposed structure of SENP1 is evolutionarily conserved with certain patches of amino acids showing even higher degree of conservation. This indicates that these patches/regions are important for interactions with other macromolecules. One big region/patch with high evolutionary conservation mapped, covers the whole face of SENP1 structure (residues 441-644) (Figure S5A). Interestingly the region that is highly conserved the most across the surface of SENP1 is also the region that was shown to be the strong UXT binding site based on the peptide array (showing binding on the longest continuous array of peptides) (Figure S5A and S4). Quantitative western analysis of the pull down assay using different deletion constructs revealed somewhat stronger binding for residues found from 297-644 (Figure 5E). Closer inspection of the structure of SENP1, the disorder prediction data (Shown in Figure S6A) and evolutionary sequence map for SENP1 pointed us to an interesting observation, where the region between residues 297-416 is mostly intrinsically disordered and showed partial high evolutionary sequence conservation (Figure S6A and S6B). Within this region, residues 297-415 are completely disordered/unstructured, whereas residues 416-466 are within the folded/structured and highly conserved region. The N-terminal disordered region described here is also supported by the recent AlphaFold prediction (See supplementary methods), where regions 1-415 are predicted as largely unstructured. These observations together with the PD-assay and the peptide array map supports the explanation that the interaction observed in the unstructured N-terminal part is through a linear epitope, whereas the structured part contributes to the interaction through a folded/3D epitope.

Supplementary Materials and Methods

Expression and purification of GST-fusion proteins

Protein overexpression in *E. coli* was induced with 0.4 mM isopropyl β-D-1-thiogalactopyranoside (IPTG) at 16 ^{0}C overnight. The cells were harvested by centrifugation at 4 ^{0}C and 4000 rpm for 15 minutes. The pellet was resuspended with a column buffer (20 mM Tris pH 8, 300 mM NaCl, 0.5 mM EDTA and 0.5 % NP-40). Lysis was achieved by freezing and thawing of the cells followed by sonication for 3× 20 seconds at 60 % amplitude. The lysed cells were spun for 30 minutes at 4 ^{0}C and 18000 rpm. Glutathione-Sepharose 4B beads (GE Healthcare) were used to bind the GST-fused recombinant proteins followed by wash with column buffer once. The beads were then washed twice with wash buffer I (20 mM Tris pH 8, 1000 mM NaCl, 0.5 mM EDTA, and 0.1 % NP-40), twice with wash buffer II (20 mM Tris pH 8, 500 mM NaCl, 0.5 mM EDTA, and 0.1 % NP-40) and once with wash buffer III (20 mM Tris pH 8, 200 mM NaCl and 0.5 mM EDTA). Bound proteins were eluted with freshly prepared elution buffer (200 mM Tris pH 8, 40 mM reduced glutathione, 200 mM NaCl, and 10% glycerol). Dialysis against dialysis buffer (30 mM Tris-HCl pH 8.0, 100 mM KCl, 5 mM $MgCl_2$, and 2 mM DTT) was carried out in Spectra/Por 1 dialysis membrane with MWCO of 6-8 kDa (Spectrum Laboratories) for 1 h at 4 ^{o}C. Protein quantitation was made using Quick Start Bradford 1× Dye Reagent (Bio-Rad).

ChIP-Seq analysis

The quality of raw sequencing reads was investigated using fastqc (version 0.11.2) (Andrews, S., *FastQC: a quality control tool for high throughput sequence data. Available online at: http://www.bioinformatics.babraham.ac.uk/projects/fastqc.* 2010). Low quality reads and adaptors were removed using TrimGalore (version 0.3.3) (Krueger, F., *Trim Galore! Available online at: http://www.bioinformatics.babraham.ac.uk/projects/trim_galore/*). Reads aligning to PhIX sequence (RefSeq: NC 001422.1) were removed using BBMap (version 37.4) (Bushnell, B., *BBMap short read aligner, and other bioinformatic tools. Available online at: http://bbmap.sourceforge.net/.* 2010). Processed reads were aligned to the human reference genome assembly, hg19 using the bwa-mem algorithm in Burrows-Wheeler-Aligner (version 0.7.5a) [13]. Aligned reads with poor mapping quality (MAPQ < 20), PCR duplicates, ambiguously aligned reads, and reads that mapped to the mitochondrial chromosome were filtered using SAMtools (version 1.3.1) [14].

Peaks were generated using MACS2 (version 2.1.0) [15], narrow peaks were called using the parameters "-g hs -m 5 50 --bw 150 --fix-bimodal --extsize 100 --call-summits -q 0.01". The bdgcmp function of MACS2 was used to refine the resulting peaks using the "Poisson Pvalue" (ppois) method. The refined peaks in bedGraph format were converted to bigWig format using bedGraphToBigWig (version 4) [16], the peaks were then visualized using the UCSC genome browser [2].

Bioinformatics analysis

Homolog sequences of the human UXT were searched using PSI-BLAST within NCBI's "non-redundant" (Nr) protein database with default parameters. After convergence, organism

filtering was set to different taxa in metazoans. Amino acid sequence conservation of homolog sequences of the human UXT proteins was investigated by performing multiple sequence alignment (MSA) using the MAFFT algorithm. SENP1 homolog sequences were searched using DELTA-BLAST within Nr protein database with default parameters until convergence followed by organism filtering set to metazoans. Amino acid sequence conservation of SENP1was investigated using MSA of protein sequences using the Expresso algorithm (*www.tcofee.org*) by providing the 3D structure of SENP1 (PDB ID: 2IY1) as a template. The resulting MSAs were visualized using Jalview (v.2.10) [11]. Secondary structure and disorder predictions of SENP1 protein sequence were made using PSIPRED (v.3.3) [9, 10] and DISOPRED3 [8], respectively. Modeller (v.9.19) was used for loop modelling of SENP1 structure (PDB ID: 2IY1). Amino acid sequence conservation of SENP1 was mapped to the SENP1 protein structure by employing the Consurf Server [3-7].

Full-length SENP1 structure prediction from AlphaFold was examined from (https://alphafold.ebi.ac.uk/entry/Q9P0U3), where almost the entire N-terminal region of SENP1 (residues 1-415) are designated as unstructured. This is supported by the low/very low pLDDT (per-residue confidence score) with pLDDT between 50 and 70 or pLDDT < 50, respectively [17, 18].

References

1. Lemma, R.B., M. Ledsaak, B.M. Fuglerud, G.K. Sandve, R. Eskeland, and O.S. Gabrielsen, *Chromatin occupancy and target genes of the haematopoietic master transcription factor MYB.* Sci Rep, 2021. **11**(1): p. 9008.

2. Kent, W.J., C.W. Sugnet, T.S. Furey, K.M. Roskin, T.H. Pringle, A.M. Zahler, and D. Haussler, *The human genome browser at UCSC.* Genome Res, 2002. **12**(6): p. 996-1006.

3. Landau, M., I. Mayrose, Y. Rosenberg, F. Glaser, E. Martz, T. Pupko, and N. Ben-Tal, *ConSurf 2005: the projection of evolutionary conservation scores of residues on protein structures.* Nucleic Acids Res, 2005. **33**(Web Server issue): p. W299-302.

4. Celniker, G., G. Nimrod, H. Ashkenazy, F. Glaser, E. Martz, I. Mayrose, T. Pupko, and N. Ben-Tal, *ConSurf: Using Evolutionary Data to Raise Testable Hypotheses about Protein Function.* Israel Journal of Chemistry, 2013. **53**(3-4): p. 199-206.

5. Ashkenazy, H., E. Erez, E. Martz, T. Pupko, and N. Ben-Tal, *ConSurf 2010: calculating evolutionary conservation in sequence and structure of proteins and nucleic acids.* Nucleic Acids Res, 2010. **38**(Web Server issue): p. W529-33.

6. Glaser, F., T. Pupko, I. Paz, R.E. Bell, D. Bechor-Shental, E. Martz, and N. Ben-Tal, *ConSurf: identification of functional regions in proteins by surface-mapping of phylogenetic information.* Bioinformatics, 2003. **19**(1): p. 163-4.

7. Ashkenazy, H., S. Abadi, E. Martz, O. Chay, I. Mayrose, T. Pupko, and N. Ben-Tal, *ConSurf 2016: an improved methodology to estimate and visualize evolutionary conservation in macromolecules.* Nucleic Acids Res, 2016. **44**(W1): p. W344-50.

8. Jones, D.T. and D. Cozzetto, *DISOPRED3: precise disordered region predictions with annotated protein-binding activity.* Bioinformatics, 2015. **31**(6): p. 857-63.

9. Buchan, D.W., F. Minneci, T.C. Nugent, K. Bryson, and D.T. Jones, *Scalable web services for the PSIPRED Protein Analysis Workbench.* Nucleic Acids Res, 2013. **41**(Web Server issue): p. W349-57.

10. Jones, D.T., *Protein secondary structure prediction based on position-specific scoring matrices.* J Mol Biol, 1999. **292**(2): p. 195-202.

11. Waterhouse, A.M., J.B. Procter, D.M. Martin, M. Clamp, and G.J. Barton, *Jalview Version 2--a multiple sequence alignment editor and analysis workbench.* Bioinformatics, 2009. **25**(9): p. 1189-91.

12. Cameron, J., O.L. Holla, K.E. Berge, M.A. Kulseth, T. Ranheim, T.P. Leren, and J.K. Laerdahl, *Investigations on the evolutionary conservation of PCSK9 reveal a functionally important protrusion.* FEBS J, 2008. **275**(16): p. 4121-33.

13. Li, H. and R. Durbin, *Fast and accurate short read alignment with Burrows-Wheeler transform.* Bioinformatics, 2009. **25**.

14. Li, H., B. Handsaker, A. Wysoker, T. Fennell, J. Ruan, N. Homer, G. Marth, G. Abecasis, R. Durbin, and S. Genome Project Data Processing, *The Sequence Alignment/Map format and SAMtools.* Bioinformatics, 2009. **25**(16): p. 2078-9.

15. Zhang, Y., T. Liu, C.A. Meyer, J. Eeckhoute, D.S. Johnson, B.E. Bernstein, C. Nusbaum, R.M. Myers, M. Brown, W. Li, and X.S. Liu, *Model-based analysis of ChIP-Seq (MACS).* Genome Biol, 2008. **9**(9): p. R137.

16. Kent, W.J., A.S. Zweig, G. Barber, A.S. Hinrichs, and D. Karolchik, *BigWig and BigBed: enabling browsing of large distributed datasets.* Bioinformatics, 2010. **26**(17): p. 2204-7.

17. Varadi, M., S. Anyango, M. Deshpande, S. Nair, C. Natassia, G. Yordanova, D. Yuan, O. Stroe, G. Wood, A. Laydon, A. Zidek, T. Green, K. Tunyasuvunakool, S. Petersen, J. Jumper, E. Clancy, R. Green, A. Vora, M. Lutfi, M. Figurnov, A. Cowie, N. Hobbs, P. Kohli, G. Kleywegt, E. Birney, D. Hassabis, and S. Velankar, *AlphaFold Protein Structure Database: massively expanding the structural coverage of protein-sequence space with high-accuracy models.* Nucleic Acids Res, 2022. **50**(D1): p. D439-D444.

18. Jumper, J., R. Evans, A. Pritzel, T. Green, M. Figurnov, O. Ronneberger, K. Tunyasuvunakool, R. Bates, A. Zidek, A. Potapenko, A. Bridgland, C. Meyer, S.A.A. Kohl, A.J. Ballard, A. Cowie, B. Romera-Paredes, S. Nikolov, R. Jain, J. Adler, T. Back, S. Petersen, D. Reiman, E. Clancy, M. Zielinski, M. Steinegger, M. Pacholska, T. Berghammer, S. Bodenstein, D. Silver, O. Vinyals, A.W. Senior, K. Kavukcuoglu, P. Kohli, and D. Hassabis, *Highly accurate protein structure prediction with AlphaFold.* Nature, 2021. **596**(7873): p. 583-589.
